# Meis1 specifies positional information in the retina and tectum to organize the zebrafish visual system

**DOI:** 10.1186/1749-8104-5-22

**Published:** 2010-09-01

**Authors:** Timothy Erickson, Curtis R French, Andrew J Waskiewicz

**Affiliations:** 1 Department of Biological Sciences, University of Alberta, CW405, Biological Sciences Bldg, Edmonton T6G 2E9, Canada; 2 Centre for Neuroscience, University of Alberta, CW405, Biological Sciences Bldg, Edmonton T6G 2E9, Canada; 3 Women and Children's Health Research Institute, University of Alberta, CW405, Biological Sciences Bldg, Edmonton T6G 2E9, Canada

## Abstract

**Background:**

During visual system development, multiple signalling pathways cooperate to specify axial polarity within the retina and optic tectum. This information is required for the topographic mapping of retinal ganglion cell axons on the tectum. Meis1 is a TALE-class homeodomain transcription factor known to specify anterior-posterior identity in the hindbrain, but its role in visual system patterning has not been investigated.

**Results:**

*meis1 *is expressed in both the presumptive retina and tectum. An analysis of retinal patterning reveals that Meis1 is required to correctly specify both dorsal-ventral and nasal-temporal identity in the zebrafish retina. Meis1-knockdown results in a loss of *smad1 *expression and an upregulation in *follistatin *expression, thereby causing lower levels of Bmp signalling and a partial ventralization of the retina. Additionally, Meis1-deficient embryos exhibit ectopic Fgf signalling in the developing retina and a corresponding loss of temporal identity. Meis1 also positively regulates *ephrin *gene expression in the tectum. Consistent with these patterning phenotypes, a knockdown of Meis1 ultimately results in retinotectal mapping defects.

**Conclusions:**

In this work we describe a novel role for Meis1 in regulating Bmp signalling and in specifying temporal identity in the retina. By patterning both the retina and tectum, Meis1 plays an important role in establishing the retinotectal map and organizing the visual system.

## Background

In order to preserve the spatial coordinates of visual input, retinal ganglion cell (RGC) axons are topographically organized in the visual processing centres of the midbrain. Retinotopic mapping has been most extensively studied in the optic tectum of fish, amphibians, and chick, and in the superior colliculus of mice. Within both the retina and the tectum, axially restricted expression of the Eph and Ephrin families of axon guidance molecules provides some of the positional information required for retinotectal map formation. Interactions between Eph receptor tyrosine kinases and their cognate Ephrin ligands result in cytoskeletal rearrangements and changes in cell adhesion, thereby eliciting either repulsive or attractive responses. By interpreting the molecular Eph and Ephrin code, RGC axons form a precisely ordered arrangement within the optic tectum that accurately reflects their axial position within the retina [1,2].

Axial patterning of the retina is required to establish the correct domains of *Eph *and *Ephrin *expression. During eye development, retinal patterning occurs along both the dorsal-ventral (DV) and nasal-temporal (NT) axes [3,4]. The DV axis is established through an antagonistic relationship between the Bone morphogenetic protein (Bmp) and Hedgehog signalling pathways. In the dorsal retina, Smad-dependent Bmp/Growth differentiation factor (Gdf) signalling initiates expression of the dorsal-specific T-box transcription factors *tbx5 *and *tbx2b*, which in turn activate *ephrinB *expression [5-8]. Additionally, Wnt signalling is required to maintain dorsal identity [9,10]. In the ventral retina, Hedgehog signals from the ventral midline induce the expression of Vax homeodomain transcription factors [11-13], thereby establishing ventral *ephB *expression [14-17]. Restricted *ephrinB *and *ephB *expression along the DV axis is required for normal formation of the retinotectal map [18,19].

The NT axis is defined by the restricted expression of forkhead transcription factors *foxG1 *(*bf1*) and *foxD1 *(*bf2*) in the nasal and temporal retina, respectively [20-22]. These factors function antagonistically to promote the expression of *ephrinA *ligands in the nasal retina and a subset of *ephA *receptors in the temporal domain. Altering the normal domains of *ephrinA *and *ephA *expression causes defects in retinotectal map formation [23-25]. Similarly to the DV axis, secreted signalling proteins are also involved in NT patterning. Fibroblast growth factor (Fgf) signals from the telencephalon and periocular mesenchyme promote nasal (*ephrinA*) and repress temporal fates (*ephA*) [26-28]. At this time, it is not clear whether temporal identity represents a retinal ground state or if it is induced by an unidentified factor.

Axial patterning of the tectum/superior colliculus is also a critical component of proper retinotectal map formation. In the midbrain, *eph *and *ephrin *genes are expressed in opposing gradients. EphrinA ligands are expressed in a posterior to anterior gradient, while EphA receptors are expressed in an opposing anterior to posterior gradient [29]. Likewise, along the medial-lateral axis, EphrinB ligands tend to be expressed in a medial to lateral gradient while EphB receptors exhibit an opposing lateral to medial gradient [19]. These opposing gradients, together with the repulsive interactions between Eph-Ephrin molecules, suggested a gradient matching model of retinotectal map formation [30]. This model is supported by experiments showing that, for example, *EphA3*-expressing temporal RGCs tend not to innervate posterior regions of the tectum expressing high levels of EphrinA ligands [31,32]. However, this model does not explain all facets of retinotectal map formation, and other factors such as attractive Eph-Ephrin interactions, axon competition [33], and other molecular cues may refine the process [30]. It is clear, however, that the precise topographic mapping of RGCs onto the tectum/superior colliculus is a highly regulated process in which Eph and Ephrin interactions play a key role.

Eph and Ephrin interactions have been well studied in the hindbrain, where they are involved in cell sorting and restricting cell movements between rhombomeres [34-36]. Of particular importance in regulating hindbrain *eph *and *ephrin *expression are the TALE-class homeodomain transcription factors Meis/Pknox and Pbx, which act in trimeric complexes with Hox proteins to impart segmental identity to the hindbrain rhombomeres [37-41]. However, Pbx and Meis also perform Hox-independent roles in eye, lens, midbrain, heart and muscle development [42-46].

Meis1 is a particularly attractive candidate for playing an important role in patterning the visual system. *meis1 *expression in the developing eye and midbrain is conserved across multiple species, and Meis1-deficiency causes microphthalmia in mice, chickens and zebrafish [47-49]. The *Drosophila *Meis homolog Homothorax (Hth) also plays an important role in insect eye development [50,51]. Structurally, Meis proteins contain a Pbx-interaction domain in the amino terminus, a DNA-binding homeodomain and a carboxy-terminal activation domain [52]. In addition to the trimeric Meis-Pbx-Hox complexes that regulate hindbrain patterning, Meis proteins can form heterodimeric complexes with Pbx and with a subset of posterior Hox proteins [53,54]. Meis and its binding partners have been identified as important regulators of *eph *and *ephrin *gene expression in the midbrain and hindbrain through both direct and indirect mechanisms [37,44,55-59]. However, despite this well-characterized role in hindbrain axial patterning and the regulation of *eph *and *ephrin *gene expression, the function of Meis1 in axial patterning of the retina and in the formation of the retinotectal map has not been fully addressed.

In this study, we use morpholino-mediated knockdown of Meis1 protein in zebrafish to determine if Meis1 patterns the retinotectal system. In the DV axis, Meis1 promotes ocular Bmp signalling through the positive regulation of *smad1 *expression and the negative regulation of *follistatin a *(*fsta*). With regard to NT patterning, Meis1 knockdown causes a loss of temporal identity in the retina. This phenotype can be attributed to an increase in retinal Fgf signalling and a decrease in *foxd1 *expression in the temporal retina. We also demonstrate that Meis1 positively regulates *ephrin *gene expression in the tectum. Consistent with these patterning defects, Meis1-depleted embryos also exhibit retinotectal mapping defects in both the NT and DV axes. We conclude that Meis1 contributes to retinotectal map formation by specifying positional information in both the retina and tectum.

## Results

### *meis1 *expression and morpholino knockdown

**Figure 1:**
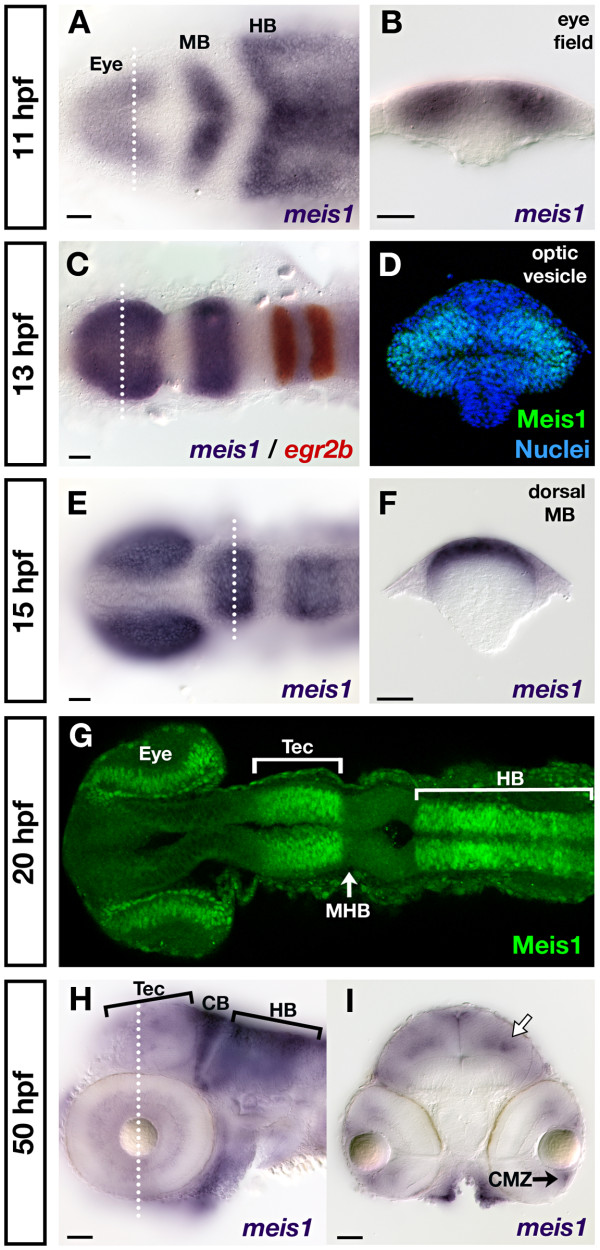
**Developmental time course of *meis1 *mRNA and protein expression**. **(A, B) **mRNA *in situ *hybridizations (ISHs) for *meis1 *at 11 hpf showing expression in the eye field, and in the presumptive midbrain (MB) and hindbrain (HB). The transverse section (B) shows *meis1 *expression in the eye field. **(C) **mRNA ISH showing *meis1 *expression at 13 hpf in the optic vesicles, midbrain and hindbrain. *egr2b*/*krox20 *expression (red) marks rhombomeres 3 and 5 of the hindbrain. **(D) **Transverse section of a whole mount immunostain for Meis1 showing protein in the dorsal and ventral leaflets of the 13 hpf optic vesicle. Hoechst 33258 stain marks the nuclei. **(E, F) **mRNA ISH at 15 hpf showing continued expression of *meis1 *in the optic vesicles, midbrain and hindbrain. The transverse section (F) shows *meis1 *expression in the dorsal midbrain. **(G) **Whole mount immunostain for Meis1 protein at 20 hpf. Meis1 protein is present in the eye, presumptive tectum (Tec), and in the hindbrain (HB) up to the r1-r2 boundary. Meis1 is excluded from the midbrain-hindbrain boundary (MHB). **(H, I) **mRNA ISH at 50 hpf showing *meis1 *expression in the hindbrain (HB) and cerebellum (CB) and tectum (Tec). The transverse section (I) shows *meis1 *expression in the ciliary marginal zone (CMZ) of the retina and in the dorsal midline and a deeper layer of the tectum (white arrow). Embryos in (A, C, E, G) are shown in dorsal view with anterior to the left. Embryo in (H) is shown in lateral view with anterior to the left. Transverse sections in (B, D, F, I) are oriented dorsal up. The dotted lines in (A, C, E, H) indicate the position of the corresponding transverse sections in (B, D, F, I). All scale bars = 50 μm.

Zebrafish *meis1 *is expressed in the presumptive eye, midbrain and hindbrain regions between 11 and 15 hours post-fertilization (hpf; Figure 1A-F) [38]. A transverse section through the optic vesicle of a 13 hpf embryo stained by a Meis1 monoclonal antibody reveals the presence of Meis1 protein in both the dorsal and ventral leaflets of the eye (Figure 1D). At 15 hpf, *meis1 *mRNA is expressed in the dorsal midbrain that will go on to form the optic tectum (Figure 1F). At 20 hpf, Meis1 protein is present in the retinal progenitor cells, in the presumptive tectum, and in the hindbrain (Figure 1G). By 50 hpf, the early pattern of *meis1 *mRNA expression has changed dramatically. *meis1 *is robustly expressed in the hindbrain and cerebellum (Figure 1H), but its tectal expression has retreated to the dorsal midline and to a deeper layer of the tectum (Figure 1I). In the retina, *meis1 *expression is largely restricted to the ciliary marginal zone (CMZ; Figure 1I). The robust expression of *meis1 *in the eye and tectum at early developmental stages (10 to 20 hpf) suggests that Meis1 may be playing an early role in patterning the zebrafish visual system.

To examine the function of Meis1 in eye and midbrain development, we used an ATG-targeted translation-blocking morpholino to knock down Meis1 protein expression [44]. This morpholino was used in all experiments unless otherwise noted. To determine the effectiveness of this morpholino, we compared the levels of Meis1 protein between 16 hpf wild-type and *meis1 *morphant embryos by whole-mount immunohistochemistry using a monoclonal antibody against zebrafish Meis1 (Additional file 1A, B). In *meis1 *morphant embryos, the specific Meis1 signal is lost, showing that the morpholino effectively reduces Meis1 protein levels. We also observe a similar knockdown of Meis1 protein using a second, non-overlapping translation blocking morpholino (*meis1*NOL; Additional file 1C, D). Furthermore, *meis1*NOL gives similar retinal patterning phenotypes to the ATG morpholino (Additional file 1E-H). Together, these results suggest that the *meis1 *morpholino represents an accurate Meis1 loss of function model.

### Meis1 knockdown results in downregulation of *ephrin *gene expression in the tectum

**Figure 2:**
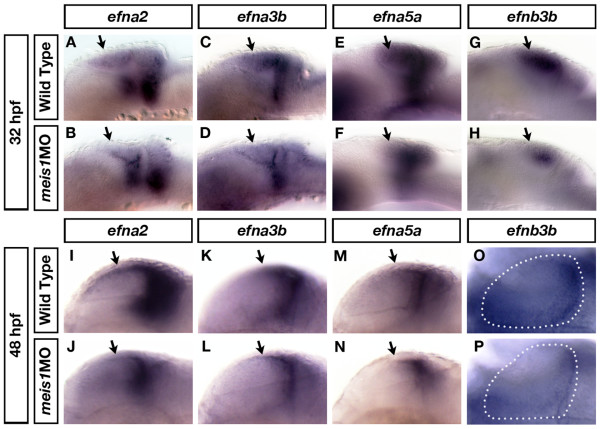
**Meis1 positively regulates *ephrin *gene expression in the tectum**. **(A-H) **mRNA *in situ *hybridizations (ISHs) for *efna2 *(A, B), *efna3b *(C, D), *efna5a *(E, F) and *efnb3b *(G, H) in 32-hpf wild-type (A, C, E, G) and *meis1 *morphant (*meis1*MO) (B, D, F, H) embryos. Meis1 knockdown leads to a downregulation in the tectal expression of these *ephrin *genes (arrows) **(I-P) **mRNA ISH for *efna2 *(I, J), *efna3b *(K, L), *efna5a *(M, N) and *efnb3b *(O, P) in 48-hpf wild-type (I, K, M, O) and *meis1 *morphant (J, L, N, P) embryos. The early defects in tectal *ephrin *gene expression remain in 48-hpf *meis1 *morphants (arrows). The dotted lines in (O, P) outline a single tectal lobe in each embryo. Embryos in (A-N) are shown in lateral view with anterior left, while embryos in (O, P) are shown in dorsal view with anterior left.

Axial patterning of the tectum is an important element in retinotectal map formation. Precise patterns of *eph *and *ephrin *expression within the tectum establish positional cues that, together with the *eph *and *ephrin *genes expressed in the retina, instruct the innervation patterns of the RGC axons. Since Meis1 is expressed in the developing tectum, and Meis proteins have been shown to regulate *eph *and *ephrin *expression in the midbrain [56,57,59], we tested whether zebrafish Meis1 also plays a critical role in tectal patterning by examining the expression of *ephrin *genes. In 32 hpf embryos, *ephrin A2 *(*efna2*), *efna3b*, *efna5a *and *efnb3b *are all expressed in the presumptive tectum, with the *ephrinA *genes also being expressed in a DV domain at the midbrain-hindbrain boundary and cerebellum (Figure 2A, C, E, G). In *meis1 *morphants, the tectal expression of *efna2 *(*n *= 11/11), *efna3b *(*n *= 13/13), *efna5a *(*n *= 57/57), and *efnb3b *(*n *= 12/14) are reduced (Figure 2B, D, F, H). These defects in *ephrin *gene expression are not due to failings in midbrain-hindbrain boundary formation as *fgf8a *(*n *= 35/35), *eng2a *(*n *= 31/31), and *pax2a *(*n *= 18/18) expression at the midbrain-hindbrain boundary is normal in *meis1 *morphants (Additional file 2). Together, these results suggest that Meis1 positively contributes to *ephrin *gene expression in the presumptive tectum.

By 48 hpf, the tectum has adopted a more mature morphology and the RGC axons have started to innervate their target zones [60]. Therefore, we examined *ephrin *gene expression in *meis1 *morphants again at this developmental stage. Consistent with the results obtained at 32 hpf, the expression levels of *efna2 *(*n *= 23/25), *efna3b *(*n *= 16/19), *efna5a *(*n *= 26/30), and *efnb3b *(*n *= 13/13) are reduced in Meis1-depleted embryos (Figure 2I-P). Taken together, these results suggest that Meis1 is required for proper tectal patterning, a role that may contribute to the retinotopic organization of the zebrafish visual system.

### Meis1 knockdown affects early retinal DV patterning and results in a partial ventralization of the retina

**Figure 3:**
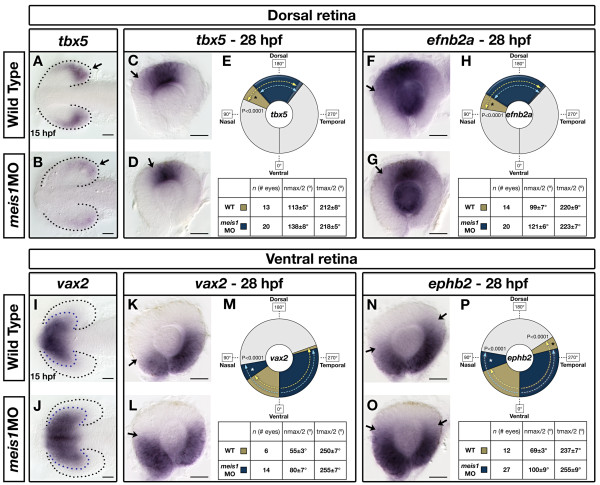
**Meis1 contributes to dorsal-ventral patterning in the developing retina**. **(A, B, I, J) **mRNA *in situ *hybridization (ISH) for the dorsal marker *tbx5 *(A, B) and the ventral marker *vax2 *(I, J) in 15-hpf wild-type and *meis1 *morphant (*meis1*MO) embryos. Black dotted lines outline the optic vesicle. Arrows indicate *tbx5 *expression in the presumptive dorsal retina. Purple dotted lines indicate the domain of *vax2 *expression. All views are dorsal with anterior left. **(C, D, F, G, K, L, N, O) **mRNA ISH for dorsal genes *tbx5 *(C, D) and *efnb2a *(F, G), and ventral genes *vax2 *(K, L) and *ephb2 *(N, O) in dissected, flat-mounted eyes from 28-hpf wild-type and *meis1 *morphant embryos. Arrows indicate the approximate limit of the gene expression domain. **(E, H, M, P) **The domains of gene expression were quantified by determining a 360° profile of *in situ *staining intensity and graphing the radial position at which gene expression intensity falls to the halfway point between its minimum and maximum values. The nmax/2 and tmax/2 values are given as the mean radial position in degrees ± one standard deviation. Asterisks indicate regions in which there are statistically significant differences in axial identity between wild type (WT) and *meis1 *morphants as determined by an unpaired, two-tailed *t*-test using a *P*-value of 0.01 as a cutoff for significance. Representative dissected eyes are shown. Scale bars = 50 μm.

The role of Meis1 in patterning the retina has not been previously examined. To determine if Meis1 is involved in specifying DV identity in the retina, we compared the expression of the DV markers *tbx5 *and *vax2 *between wild-type and Meis1-depleted embryos. At 15 hpf, *tbx5 *is expressed in the presumptive dorsal retina (Figure 3A). Knockdown of Meis1 reduces both the domain and intensity of *tbx5 *expression (*n *= 27/30; Figure 3B). To examine the domain of ventral identity at 15 hpf, we looked at the expression of *vax2*. Compared to wild-type embryos, the retinal domain of *vax2 *expression is expanded upon Meis1 knockdown (*n *= 14/19; Figure 3I, J). Taken together, these data suggest that a loss of Meis1 function results in a reduction in presumptive dorsal retinal identity together with an expansion of ventral identity.

To determine if these early defects in DV patterning persist into later eye development, we examined dorsal *tbx5 *and *efnb2a *and ventral *vax2 *and *ephb2 *expression in 28-hpf retinas. By this stage, the wild-type zebrafish eye has adopted a more definitive morphology where the neural retina wraps around the lens and meets at the ventral choroid fissure to form a 360° circle. To quantify changes in retinal axial patterning, we analyzed *in situ *staining intensity in flat-mounted retinas from 28-hpf wild-type and *meis1 *morphants and compared the radial position at which gene expression intensity falls to the halfway point between its minimum and maximum values (see Materials and methods) [27]. Consistent with the defects observed at 15 hpf, we find that the extent of *tbx5 *expression is reduced in 28-hpf *meis1 *morphants (Figure 3C-E). This reduction in *tbx5 *is primarily at its dorso-nasal boundary, where *meis1 *morphants exhibit a 26° retraction in expression (*P *< 0.0001; Figure 3E). In contrast, the dorso-temporal border of *tbx5 *does not statistically differ between wild type and *meis1 *morphants (*P *= 0.0124; Figure 3E). *efnb2a *is a transcriptional target of *tbx5 *in the dorsal retina. Similar to the changes in *tbx5 *expression, we find that the dorso-nasal border of *efnb2a *is retracted by 22° in *meis1 *morphants (*P *< 0.0001), and the dorso-temporal border is unchanged (*P *= 0.3301; Figure 3F-H). With regard to ventral identity at 28 hpf, we find that the ventro-nasal borders of both *vax2 *and *ephb2 *are expanded dorsally by 25° and 31°, respectively, in *meis1 *morphants (*P *< 0.0001; Figure 3K-P). While the ventro-temporal border of *vax2 *is not statistically different between wild type and morphants (*P *= 0.2046), the ventro-temporal border of *ephb2 *is retracted ventrally by 18° in *meis1 *morphants (*P *< 0.0001; Figure 3P). Overall, these results demonstrate that Meis1 plays a role in specifying DV identity, and that Meis1 knockdown leads to a partial ventralization of the retina, particularly in the ventro-nasal domain.

### Meis1 promotes retinal Bmp signalling by regulating *smad1 *and *follistatin a *expression

**Figure 4:**
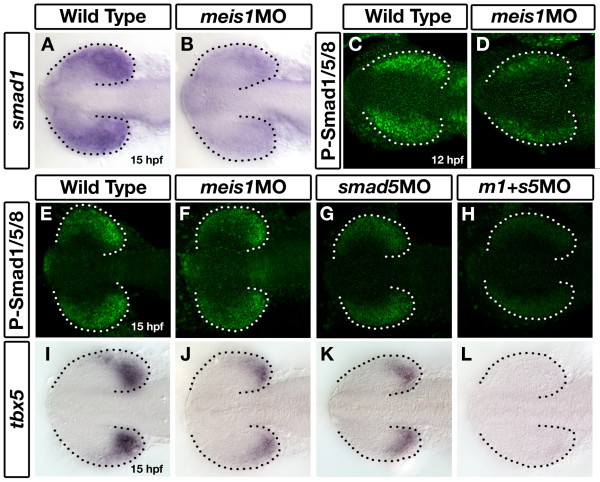
**Meis1 positively regulates *smad1 *expression in the developing eye**. **(A, B) **mRNA *in situ *hybridization (ISH) for *smad1 *in 15-hpf wild-type and *meis1 *morphant (*meis1*MO) embryos. **(C, D) **Whole mount immunostains for phosphorylated Smad1/5/8 on 12-hpf wild-type and *meis1 *morphant embryos. **(E-H) **Phospho-Smad1/5/8 immunostains on 15-hpf embryos treated with *meis1 *morpholino (F), *smad5 *morpholino (G) or a combination of both morpholinos (H). **(I-L) **mRNA ISH for *tbx5 *in 15-hpf wild-type (I), *meis1 *morphant (J), *smad5 *morphant (K), and *meis1*-*smad5 *double morphant (L) embryos. Dotted lines outline the optic vesicle. All views are dorsal with anterior to the left.

The Bmp signalling pathway plays an evolutionarily conserved role in specifying dorsal identity in the retina [5-8]. The DV patterning defects in *meis1 *morphants could be due to misregulation of a vital component of the Bmp pathway. Smad transcription factors play an essential role in this process by mediating the transcriptional response to Bmp signalling. We hypothesized that Meis1 might regulate retinal s*mad1 *expression since its domain in the early zebrafish optic vesicle is similar to that of Meis1 protein, and *meis1 *mRNA expression precedes that of *smad1 *(Additional file 3A-D). To test this hypothesis, we compared *smad1 *expression between wild type and *meis1 *morphants at 15 hpf and found that *smad1 *expression is strongly downregulated in the retina of Meis1-depleted embryos (*n *= 65/65; Figure 4A, B). This phenotype can be rescued by the co-injection of morpholino-insensitive *myc*-*meis1 *mRNA (*n *= 14/15; Additional file 4A-D), demonstrating the specificity of the *meis1 *morpholino phenotype. Furthermore, *gdf6a *morphants have normal levels of *smad1 *transcript in the immature eye at 13 hpf (Additional file 5A, B), demonstrating that early *smad1 *transcription is not regulated by Bmp signalling. Taken together, these data suggest that Meis1 is a specific regulator of *smad1 *transcription.

The phosphorylation of Smads 1, 5, and 8 by type I Bmp receptors is an essential step in transducing the Bmp signal into a transcriptional response. To see if the downregulation of *smad1 *expression had an effect on the total amount of phosphorylated Smads in the retina, we performed whole mount immunohistochemistry using a phospho-Smad1/5/8-specific antibody. At 12 hpf, phospho-Smad staining is reduced in Meis1-depleted embryos (*n *= 17/21; Figure 4C, D). Taken together with the downregulation of *tbx5 *in Meis1-depleted embryos (Figure 3A, B), these data suggest that Meis1 has a positive effect on the level of Bmp signalling during early retinal patterning.

Although the expression of *smad1 *remains downregulated in *meis1 *morphants, the reduced level of phospho-Smad1/5/8 at 12 hpf largely recovers by 15 hpf (compare Figures 4E and 4F). The presence of other Smad proteins could account for this discrepancy. *smad5 *is ubiquitously expressed during early development, can act redundantly with *smad1 *[61-63], and is not transcriptionally regulated by Meis1 (Additional file 6). To determine if the presence of Smad5 is masking the loss of *smad1 *expression in Meis1-depleted embryos at 15 hpf, we performed an interaction experiment using *meis1 *and *smad5 *morpholinos. Using the level of phospho-Smads1/5/8 and *tbx5 *transcription as an assay for Smad5 function, we observe a decrease in the level of retinal Bmp signalling in *smad5 *morphants. Knocking down Smad5 protein subtly decreases the level of phospho-Smads1/5/8 (*n *= 4/4; compare Figures 4E and 4G), and reduces *tbx5 *to levels comparable to that of Meis1-depleted embryos (*n *= 34/34; compare Figures 4K with 4I and 4J). However, by combining the two morpholinos, there is a synergistic effect where the level of phospho-Smads1/5/8 is nearly eliminated (*n *= 6/6; Figure 4H) and *tbx5 *transcript is often undetectable by *in situ *hybridization (*n *= 28/39; Figure 4L). These results are consistent with the hypothesis that Meis1-regulated transcription of *smad1 *is important for retinal DV patterning, and that Smad1 and Smad5 perform at least partially redundant functions in the eye.

**Figure 5:**
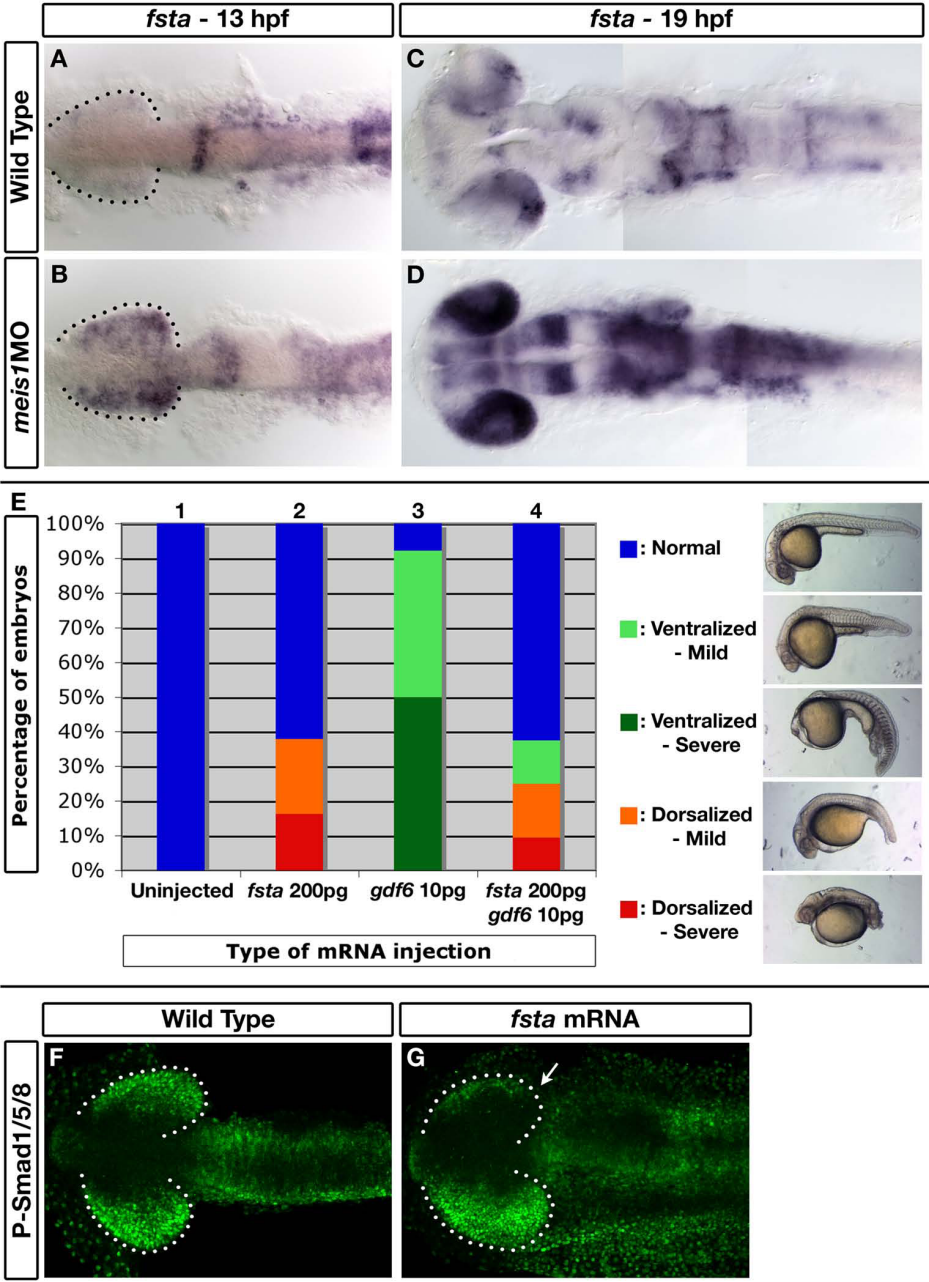
***follistatin a *is ectopically expressed in Meis1-depleted embryos and can inhibit Gdf6-mediated Bmp signalling**. **(A-D) **mRNA *in situ *hybridizations for *fsta *on 13-hpf (A, B) and 19-hpf (C, D) wild-type and *meis1 *morphant (*meis1*MO) embryos. Dotted lines outline the optic vesicle. All views are dorsal with anterior to the left. **(E) **Results of the GDF6-Fsta interaction experiments. One-cell embryos were injected with either 200 pg of zebrafish *fsta *mRNA (bar 2), human *GDF6 *mRNA (bar 3), or both mRNAs (bar 4), raised until 28 hpf, and scored for dorsalized and ventralized phenotypes (see legend on the right for classification). **(F, G) **Confocal images of whole mount immunostains for phospho-Smad1/5/8 in wild-type and *fsta *mRNA-injected embryos at 14 hpf. Injection of *fsta *mRNA into one cell of a two-cell embryo causes a unilateral reduction in phospho-Smad1/5/8 staining (arrow in G). Dotted lines outline the optic vesicle. Views are dorsal with anterior to the left.

In addition to positive regulators of retinal Bmp signalling, we also examined the role of Meis1 in regulating the expression of Bmp inhibitors. In particular, we observe that Meis1 knockdown results in an upregulation of *fsta *expression throughout much of the brain and anterior spinal cord (*n *= 48/48; Figure 5A-D; Additional file 1G, H). Especially striking is the ectopic *fsta *expression in the retina at 13 hpf (Figure 5B). As with *smad1*, injection of *myc-meis1 *mRNA can partially rescue the *fsta *expression defects in *meis1 *morphants (*n *= 7/10; Additional file 4E-H). Additionally, this phenotype cannot be attributed to a downregulation of Bmp signalling, as *gdf6a *morphants do not exhibit increased *fsta *expression at 13 hpf (Additional file 5C, D). These data suggest that, in addition to positively regulating *smad1 *transcription, Meis1 is also a negative regulator of *fsta *expression.

Fsta is a secreted protein known to bind directly to several different Bmp ligands to prevent Bmp receptor activation [64-66]. Although Fsta can downregulate Gdf6 transcription in *Xenopus *animal caps [67], a functional antagonism between Fsta and Gdf6 proteins has not been demonstrated. To determine if the upregulation of *fsta *expression in *meis1 *morphants can inhibit *gdf6a *function in the retina, we tested the ability of ectopic *fsta *to inhibit the embryonic ventralization phenotype caused by the injection of human *GDF6 *mRNA into one-cell embryos (Figure 5E). As little as 10 pg of *GDF6 *mRNA is sufficient to cause a ventralized phenotype in 92% of the embryos, with 50% of embryos lacking all anterior head structures (*n *= 38; Figure 5E, bar 3). Conversely, 200 pg of *fsta *mRNA alone causes a dorsalized phenotype in 38% (*n *= 37) of the injected embryos (Figure 5E, bar 2). Injecting 200 pg of *fsta *mRNA together with 10 pg of *GDF6 *mRNA effectively inhibits the ventralizing effects of *GDF6 *(Figure 5E, bar 4). Following this treatment, no severely ventralized embryos were observed, and only 14% had a mildly ventralized phenotype (*n *= 32). To test if *fsta *can inhibit endogenous Gdf6a signalling in the zebrafish retina, we injected 100 pg of *fsta *mRNA into a single cell of two-cell embryos and examined the level of phospho-Smads1/5/8 at 14 hpf by whole-mount immunohistochemistry. This asymmetrical injection of *fsta *mRNA causes uniocular reductions of phospho-Smads1/5/8 (*n *= 5/8; Figure 5F, G). Together, these results suggest that Fsta can inhibit Gdf6a-mediated signalling, and that the upregulation of *fsta *expression in *meis1 *morphants may contribute to the retinal DV patterning defects observed in these embryos.

In summary, Meis1 knockdown causes a dorsal-to-ventral shift in retinal identity that correlates with a reduced level of Bmp signalling in the optic vesicle. This decreased Bmp signal in *meis1 *morphants can be attributed to a loss of *smad1 *expression and an upregulation of *fsta*. Thus, Meis1 plays an important role in retinal DV patterning by facilitating ocular Bmp signalling.

### Meis1-knockdown causes a partial loss of temporal identity in the retina

**Figure 6:**
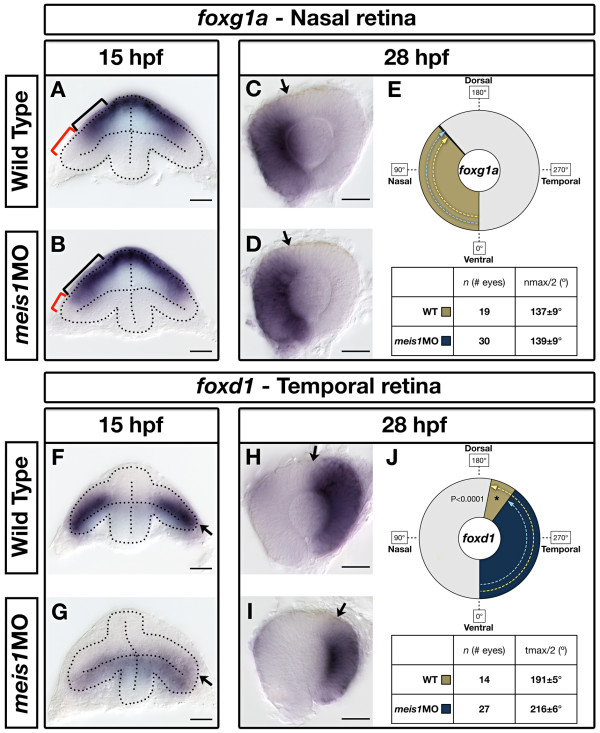
**Meis1-depleted embryos exhibit a partial temporal-to-nasal shift in retinal identity**. **(A, B, F, G) **mRNA *in situ *hybridization (ISH) for the nasal marker *foxg1a *(A, B) and the temporal marker *foxd1 *(F, G) in 15-hpf wild-type and *meis1 *morphant (*meis1*MO) embryos. Dotted lines outline the optic vesicle. The black brackets in (A, B) indicate the proximal-distal extent of *foxg1a *expression, while the red brackets indicate the *foxg1a*-free region. Arrows in (F, G) indicate the dorsal leaflet of the optic vesicle. Transverse sections are oriented dorsal up. **(C, D, H, I) **mRNA ISH for the nasal marker *foxg1a *(C, D) and the temporal marker *foxd1 *(H, I) in dissected, flat-mounted eyes from 28-hpf wild-type and *meis1 *morphant embryos. The arrows indicate the approximate limit of the gene expression domain. **(E, J) **The domains of gene expression were quantified by determining a 360° profile of *in situ *staining intensity and graphing the radial position at which gene expression intensity falls to the halfway point between its minimum and maximum values. The nmax/2 and tmax/2 values are given as the mean radial position in degrees ± one standard deviation. Asterisks indicate regions in which there are statistically significant differences in axial identity between wild type and *meis1 *morphants as determined by an unpaired, two-tailed *t*-test using a *P*-value of 0.01 as a cutoff for significance. Representative dissected eyes are shown. Scale bars = 50 μm.

During early zebrafish eye development, the nasal and temporal axes are initially established in the dorsal and ventral leaflets of the optic vesicle, respectively [28]. As the retina develops, *foxd1*-expressing cells in the ventral leaflet move into the dorsal leaflet to form the temporal domain of the neural retina. To determine if Meis1 regulates positional identity along the NT axis, we examined *foxg1a *and *foxd1 *mRNA expression in the presumptive nasal and temporal domains. In 15-hpf wild-type embryos, *foxg1a *is expressed in the dorsal leaf of the optic vesicle, specifically the proximal region fated to form the nasal retina (Figure 6A). In *meis1 *morphants, this domain of *foxg1a *expression is expanded distally, suggesting an expansion of nasal identity (*n *= 31/54; Figure 6B). Likewise, in 15-hpf wild-type embryos, *foxd1 *is also expressed in the dorsal optic vesicle, but in a domain underlying, and more distal to, that of *foxg1a *(Figure 6F). In 15 hpf *meis1 *morphants, cells in the dorsal optic vesicle do not express *foxd1 *(*n *= 44/52; Figure 6G; Additional file 1E, F). Instead, faint *foxd1 *expression is observed in the ventral leaflet, suggesting that *foxd1*-expressing cells have failed to move into the dorsal leaflet of the optic vesicle. We also examined the temporally restricted expression of *epha7 *at 16 hpf and found that its expression is similarly reduced in Meis1-depleted embryos (Additional file 7A, B). Together, these results suggest that Meis1 is an important regulator of early NT patterning.

To see how these early defects in NT patterning translate into later phenotypes, we quantified the expression domains of *foxg1a *and *foxd1 *in dissected 28-hpf retinas. At this later stage, there is no significant expansion of nasal *foxg1a *expression towards the dorsal pole in Meis1-depleted retinas (*P *= 0.5457; Figure 6C-E). Conversely, the dorso-temporal border of *foxd1 *expression is retracted ventrally by 25° in Meis1-depleted retinas (*P *< 0.0001; Figure 6H-J). Consistent with this latter observation, we also find that the expression domains of *epha7 *(*n *= 14/14) and *epha4b *(*n *= 18/20) in the temporal retina are also reduced in *meis1 *morphants (Additional file 7C-F). Together, these data support the idea that Meis1 plays a role in NT patterning, especially with regard to the establishment of *foxd1 *and *ephA *expression in the temporal retina.

### The contribution of Fgf signalling to the NT patterning defects in Meis1-depleted embryos

**Figure 7:**
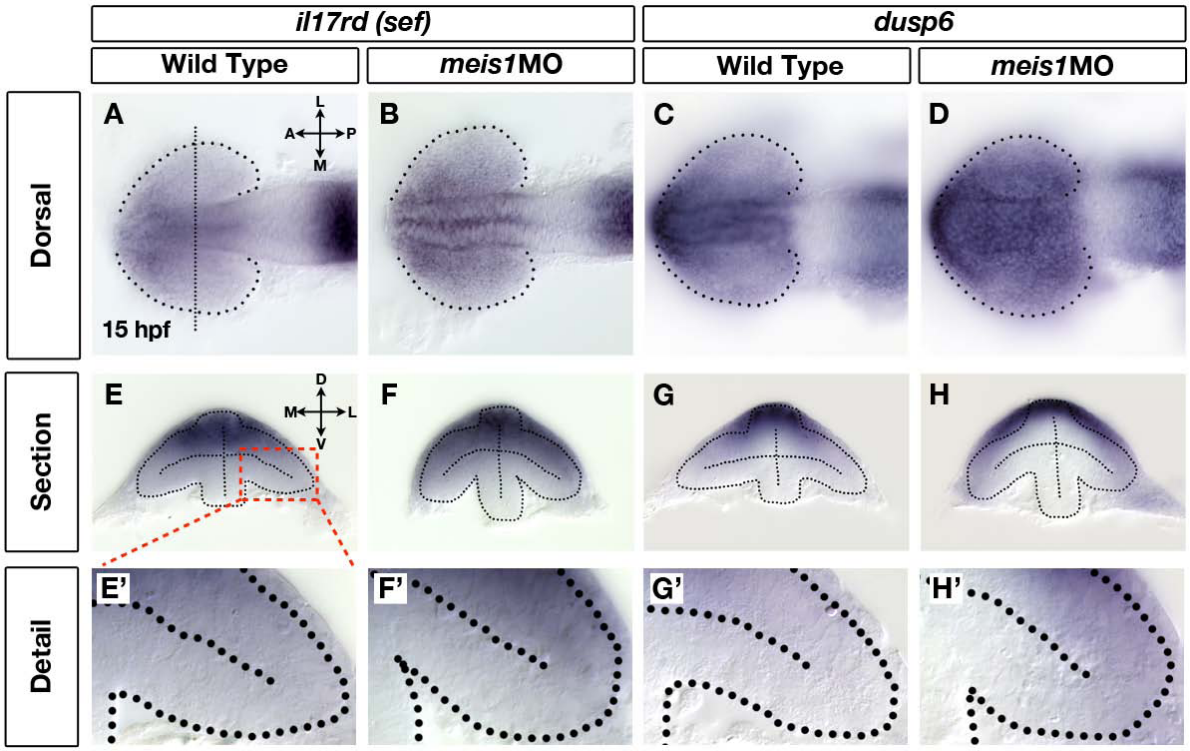
**Retinal Fgf signalling is upregulated in Meis1-depleted embryos**. **(A-D) **mRNA *in situ *hybridization (ISH) for the Fgf-responsive genes *il17rd*/*sef *(A, B) and *dusp6 *(C, D) in wild-type (A, C) and *meis1 *morphant (*meis1*MO) (B, D) embryos. Dotted lines outline the optic vesicle. The vertical dotted line in (A) indicates the estimated position of the transverse sections in (E-H). Views are dorsal with anterior left. **(E-H) **Transverse sections through the eyes of 15-hpf wild-type and *meis1 *morphant embryos stained for *il17rd *and *dusp6*. **(E'-H') **Detailed views of the corresponding sections in (E-H). The region of interest is indicated by the red dashed-line box. Dotted lines outline the optic vesicles. All transverse sections are oriented with dorsal up. Legend for retinal axial position: D, dorsal; V, ventral; N, nasal; T, temporal; L, lateral; M, medial; A, anterior; P, posterior.

The Fgf signalling pathway establishes nasal identity in the developing retina [26-28]. Ectopic Fgfs expand nasal identity at the expense of temporal fate, while inhibition of the pathway has the opposite effect. Since some aspects of the *meis1 *morphant phenotype resemble that of ectopic Fgf signalling, we examined the effect of Meis1-depletion on *il17rd*/*sef *and *dusp6 *expression, two genes whose transcription is positively controlled by the Fgf pathway [68-71]. As seen in the dorsal view, both *il17rd *(Figure 7A) and *dusp6 *(Figure 7C) are expressed in the dorsal forebrain, optic stalk and faintly in the presumptive nasal retina of 15-hpf wild-type embryos. Meis1 knockdown results in broader domains of *il17rd *(*n *= 27/48; Figure 7B) and *dusp6 *(*n *= 18/30; Figure 7D) expression in the dorsal forebrain and presumptive nasal retina. Transverse cross-sections also reveal that the nasal expression domains of *il17rd *and *dusp6 *are expanded laterally in 15-hpf Meis1-depleted embryos (*il17rd *in Figure 7E, E', F, F'; *dusp6 *in Figure 7G, G', H, H'). Thus, we can conclude from these experiments that there is a subtly higher level of Fgf signalling in the eyes and forebrain of *meis1 *morphant embryos.

**Figure 8:**
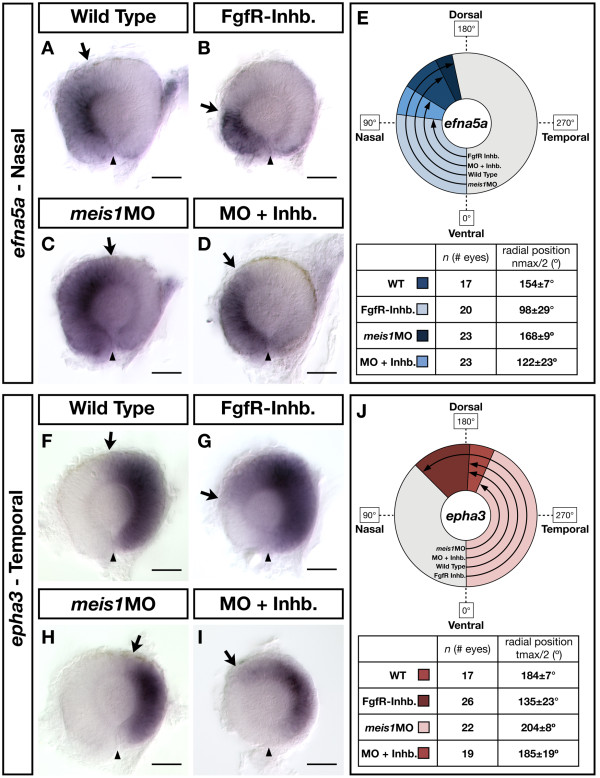
**The contribution of Fgf signalling to the NT patterning defects in Meis1-depleted embryos**. **(A-D, F-I) **mRNA *in situ *hybridizations for the NT markers *efna5a *(A-D) and *epha3 *(F-I) in wild type, Meis1-depleted (*meis1MO*), Fgf receptor-inhibitor treated (FgfR-Inhb.), and FgfR-inhibited/Meis1-depleted retinas (MO + Inhb.). Arrows indicate the extent of the gene expression domain, while the arrowheads indicate the position of the ventral choroid fissure. Representative dissected eyes are shown oriented with dorsal up and nasal to the left. Scale bars = 50 μm. **(E, J) **Quantification of the changes in *efna5a *and *epha3 *expression, as quantified by measuring a 360° profile of *in situ *staining intensity and graphing the mean radial position at which gene expression intensity falls to the halfway point between its minimum and maximum values. The nmax/2 and tmax/2 values are given as the mean radial position in degrees ± one standard deviation. WT, wild type.

To determine whether the expanded range of Fgf signalling contributes to the NT patterning defects in *meis1 *morphants, we antagonized Fgf signalling using a pharmaceutical inhibitor of Fgf receptors (PD173074). Changes in NT patterning were assayed by *in situ *hybridization for the nasal marker *efna5a *and the temporal marker *epha3 *and quantified by radial profiling of *in situ *intensity. Consistent with the NT patterning defects shown in Figure 6 and Additional file 7, *meis1 *morphants exhibit an expansion of nasal *efna5a *(mean shift of 14°; *P *< 0.0001; Figure 8A, C, E) and a reduction in temporal *epha3 *expression compared to wild type (20°; *P *< 0.0001; Figure 8F, H, J). Fgf receptor (FgfR) inhibitor-treated embryos exhibit a partial loss of nasal identity (56°; *P *< 0.0001; Figure 8B, E), and an expansion of the temporal domain (49°; *P *< 0.0001; Figure 8G, J). Compared to the *meis1 *morphant phenotype alone, morphants treated with the FgfR inhibitor exhibit reduced *efna5a *expression (46° difference; *P *< 0.0001; Figure 8D, E), and have an expanded domain of *epha3 *expression (19° difference; *P *< 0.0001; Figure 8I, J). However, neither of these phenotypes are as profound as those caused by the FgfR inhibitor treatment alone. This is true with regard to both *efna5a *and *epha3 *expression, where inhibition of Fgf signalling in *meis1 *morphants does not cause the same robust shifts in axial identity as it does in uninjected embryos. Similar results were observed in experiments using the Fgf receptor inhibitor SU5402 (data not shown). Together, these data suggest that the subtle expansion of Fgf signalling in Meis1-depleted embryos is unlikely to be the sole reason for the observed shifts in NT identity.

### Meis1 knockdown results in retinotectal map defects

**Figure 9:**
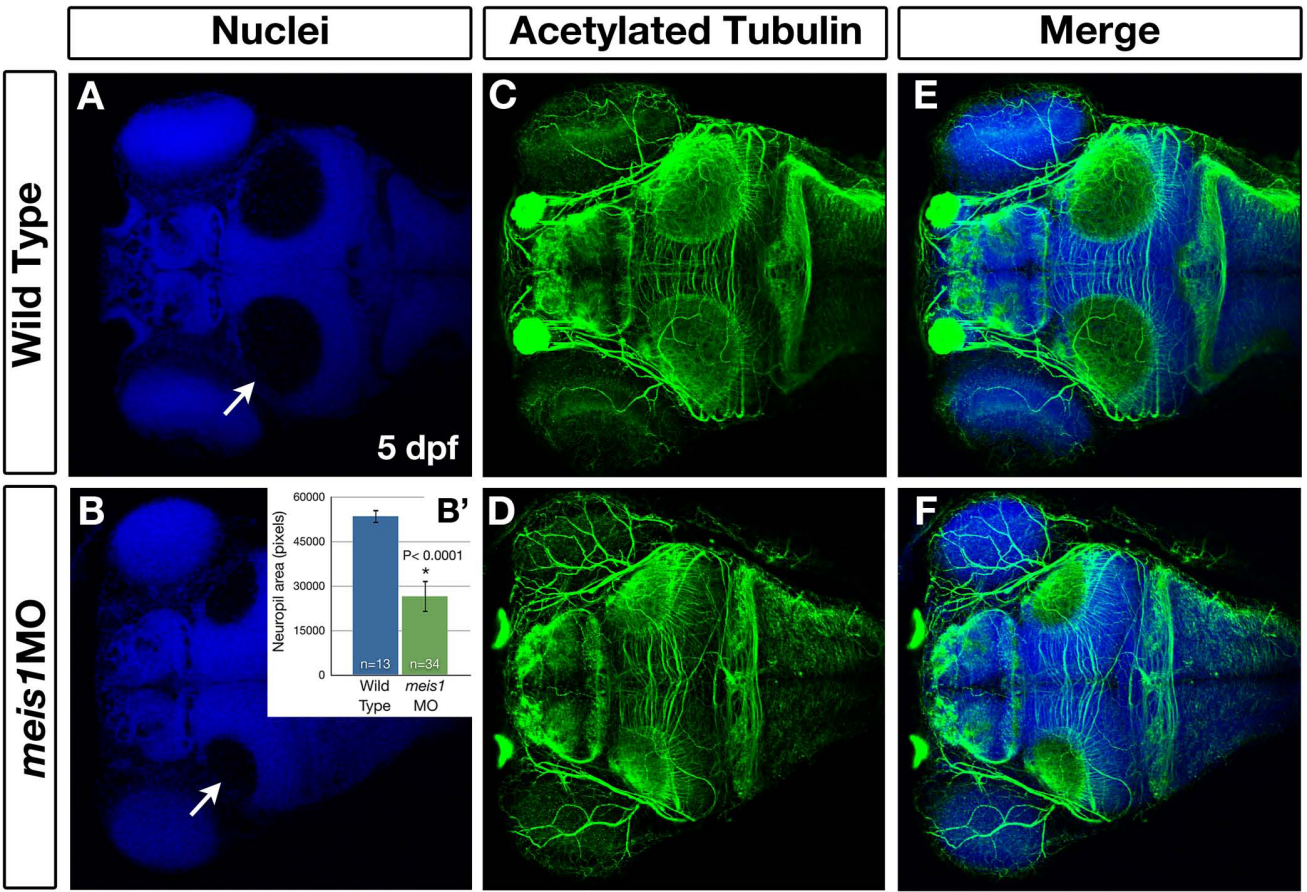
**Meis1-depleted embryos have smaller tectal neuropil at 5 dpf**. **(A-F) **Whole mount immunohistochemistry using anti-acetylated tubulin (axons - green) and Hoechst 33258 (nuclei - blue) to compare the size of the tectal neuropil in 5-dpf wild type (A, C, E) and *meis1 *morphant (*meis1*MO) (B, D, F) embryos. White arrows in (A, B) indicate the tectal neuropil. **(B') **The area (in pixels) of the neuropil from wild type and *meis1 *morphant embryos was measured using ImageJ. The *n *values represent individual neuropil regions. The error bars show plus/minus one standard deviation. The asterisk indicates a statistically significant reduction the size of morphant neuropil as determined by an unpaired, two-tailed *t*-test.

Having established that Meis1 plays an early developmental role in patterning the retina and tectum, we next determined whether this early function had a later effect on tectal development and the formation of the retinotectal map. To compare the size of the tectal neuropil, we stained 5-dpf wild-type and *meis1 *morphant embryos using an antibody against acetylated tubulin to mark axons and with Hoechst 33258 to mark nuclei (Figure 9A-F). Morphant neuropil (*n *= 34 individual neuropil) are 50% smaller on average than their wild-type counterparts (*n *= 13 individual neuropil; *P *< 0.0001; Figure 9B'). However, acetylated tubulin-positive axons are still present in morphant tecta (*n *= 19/19; Figure 9E-F), suggesting that although the tectum is smaller, its morphology is grossly normal in Meis1-depleted embryos.

**Figure 10:**
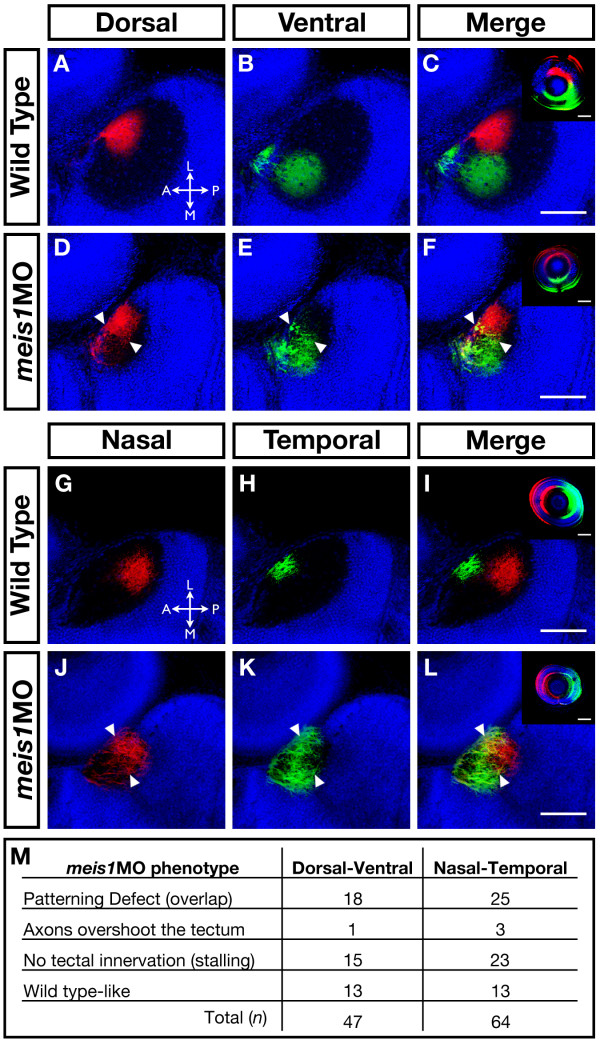
**The retinotectal map is disorganized in Meis1-depleted embryos**. **(A-L) **Lipophilic fluorescent dyes DiI (red) and DiO (green) were injected into specific axial positions of the retina of fixed 5-dpf wild-type and *meis1 *morphant embryos and innervation patterns of the ganglion cell axons on the tectum were imaged by confocal microscopy. Nuclei are stained with Hoechst 33258 (blue). The wild-type (A-C) medial-lateral segregation of dorsal (red) and ventral (green) ganglion cell axons in the tectum is lost in *meis1 *morphants (white arrowheads in (D-F)). Similarly, the wild-type (G-I) anterior-posterior segregation of nasal (red) and temporal (green) ganglion cell axons in the tectum is disorganized in *meis1 *morphants (white arrowheads in (J-L)). The insets in (C, F, I, L) are lateral views of injected retinas from the embryos shown in the corresponding panels. Retinas are oriented with dorsal up and nasal to the left, while all tectal views are dorsal with anterior to the left. Legend for axial position in the tectum: L, lateral; M, medial; A, anterior; P, posterior. All scale bars = 75 μm. **(M) **Table describing the frequency of various retinotectal mapping phenotypes observed in *meis1 *morphants (*meis1*MO). No retinotectal mapping defects were observed in any wild-type embryos examined.

To find out if Meis1-depleted embryos have retinotectal mapping defects, we injected DiI and DiO fluorescent lipophilic dyes into specific axial regions of 5-dpf retinas and visualized the mapping patterns of the RGC axons by confocal microscopy. With regard to the retinal DV axis, wild-type dorsal RGCs (red) innervate the lateral tectum, while ventral RGCs (green) project to the medial region (Figure 10A-C). Along the NT axis, wild-type nasal RGCs (red) innervate the posterior tectum, while temporal RGCs (green) project to the anterior region (Figure 10G-I). Meis1-depleted embryos exhibit defects in DV mapping, where the innervation zones of the dorsal and ventral RGC axons partially overlap in the tectum (*n *= 18/47; Figure 10D-F). Although, the normal medial-lateral restriction is lost in morphants for both dorsal and ventral axons, the ventral axons tend to exhibit a broader innervation pattern than dorsal axons (compare Figures 10B and 10E). We observe similar results with regard to the NT retinotectal map in *meis1 *morphants, in which the innervation zones of the nasal and temporal RGC axons overlap in the tectum (*n *= 25/64; Figure 10J-L). Again, while normal anterior-posterior segregation of axons is lost for both the nasal and temporal axons, temporal axons tend to be more broadly distributed in the tectum (compare Figures 10H and 10K). Although overlapping innervation patterns are a common phenotypic class in *meis1 *morphants, we also frequently observe a partial or complete axon stalling phenotype (DV, *n *= 15/47; NT, *n *= 23/64; Figure 10M; Additional file 8). These data demonstrate that Meis1 function is required to correctly organize the retinotectal map.

## Discussion

### Meis1 is required to establish tissue polarity throughout the anterior neural tube

*meis1 *is expressed in the eye field, the midbrain and hindbrain during the crucial period in which axial identities are established in these tissues. Consistent with the known role for Meis proteins in patterning the hindbrain (TE, unpublished observations) [37,38,72], in this work we demonstrate a specific role for Meis1 in establishing axial polarity in the presumptive tectum and retina. The tectum exhibits a strong anterior-posterior polarity that is reflected in opposing gradients of *eph *and *ephrin *expression [29]. In this paper, we show that Meis1 establishes polarity in the zebrafish tectum by positively regulating *efna2*, *efna3b*, *efna5a*, and *efnb3 *expression. This is consistent with what has been found in other model organisms where murine Meis2 patterns the superior colliculus by directly activating *ephA8 *expression [56] and chick Meis2 promotes tectal *ephrin B1 *expression, possibly via a direct interaction with Otx2 [59]. The functions of Meis1 in regulating *eph *and *ephrin *expression are independent of any role in midbrain-hindbrain boundary formation (Additional file 2), again consistent with Meis2 function in chick [59]. Lastly, while *meis1 *is also expressed in the developing eye, its role in patterning this tissue has not been explored. In this paper, we show that Meis1 influences the specification of the DV and NT axes in the retina. Taken together, these studies demonstrate that Meis1 contributes to the establishment of axial polarity in anterior neural tissues such as the hindbrain, midbrain and retina.

### The axial patterning roles of Meis1 contribute to formation of the retinotectal map

Meis1-depleted embryos exhibit a range of aberrant retinotectal pathfinding phenotypes, the most common of which are topographic mapping defects in the tectum (Figure 10) and axon stalling (Additional file 8). While it is clear that Meis1 plays a role in the early patterning of the presumptive DV and NT axes, these patterning phenotypes are not as robust by the time the retinal axes have assumed their final anatomical positions (Figures 3 and 6). Indeed, by 28 hpf, the axial patterning defects in *meis1 *morphants are milder than what is seen in embryos with decreased levels of Gdf6a or increased Fgf function [5,6,27,28]. However, the retinotectal mapping defects in *meis1 *morphants are more profound than expected on the basis of observed retinal patterning defects. These data suggest that the loss of Meis1 causes pleiotropic effects throughout the zebrafish visual system. Meis1 is also a regulator of retinal progenitor cell proliferation [47,48], the relevance of which to eye patterning is not yet understood. Furthermore, we observe defects in tectal size and morphology in *meis1 *morphant zebrafish (Figure 8) and an expansion of the optic stalk (Additional file 2E, F). Taken together, while it is clear that Meis1 plays a part in specifying positional information in both the retina and tectum, it is possible that this patterning role is but one way that Meis1 contributes to the organization of the retinotectal map.

### Meis1 is a positive regulator of retinal Bmp signalling

The requirement for Bmp activity in regulating dorsal retinal identity and the retinotectal map has been well established [5-8,73-76]. A decrease in Bmp signalling leads to a loss of dorsal markers such as *tbx5 *and an expansion of ventral identity as marked by *vax2*. We observe similar changes in *meis1 *morphants, suggesting that Meis1 can potentiate Bmp signalling in the retina (Figure 3). Meis1 regulates the Bmp pathway in at least two ways: first, by positively regulating *smad1 *transcription (Figure 4); and second, by repressing *fsta *expression (Figure 5). Meis proteins have not been previously characterized as positive regulators of Bmp signalling, and as such, these results point to a new role for Meis1 in the regulation of neural patterning.

Meis proteins are typically thought to act as transcriptional activators by facilitating histone acetylation at target promoters [52,77,78]. For this reason, the ectopic *fsta *expression observed in *meis1 *morphants is likely not due to Meis1 directly repressing *fsta*, although transcriptional repression by TALE-class proteins is not unheard of [79]. On the other hand, the downregulation of *smad1 *transcription in *meis1 *morphants is consistent with it being directly activated by Meis1. *meis1 *and *smad1 *are co-expressed in the presumptive retina during early development, and we observe that *smad1 *expression is never initiated properly in *meis1 *morphants. Furthermore, we have identified two putative Meis binding sites in a region upstream of the *smad1 *coding sequence that is conserved between zebrafish (*Danio rerio*), Medaka (*Oryzias latipes*), the green spotted pufferfish (*Tetraodon nigroviridis*), and the three-spined stickleback (*Gasterosteus aculeatus*) (TE, unpublished observations). Further analyses will determine whether these sites are functionally significant. Very few studies of *smad1 *transcriptional regulation have been done [80,81]; thus, the Meis1-dependent activation of *smad1 *transcription is an important finding in this area, and represents a novel mechanism of tissue-specificity in Bmp regulation.

Smads1 and 5 have been shown to act redundantly in some processes, such as bone formation and tumour suppression [61-63], yet perform distinct functions during embryonic DV patterning and blood development [82,83]. The delayed onset of Smad1/5/8 phosphorylation in *meis1 *morphants suggests that Smad1 may be especially important during the initiation of retinal Bmp signalling. However, the results of the *meis1*-*smad5 *morpholino interaction experiment suggest that Smad1 and Smad5 have partially redundant functions, at least with regard to activating *tbx5 *expression. These results suggest that Smad1 and 5 have differential and overlapping roles in patterning the zebrafish retina.

### Meis1 is an important factor in the specification of the temporal retina

Vertebrate eye patterning is characterized by a complex series of interactions in which cell identity, proliferation and eye morphogenesis [84,85] must be spatially and temporally coordinated. This has been best described with regard to nasal specification in the zebrafish retina by Fgfs 3, 8, and 24 [27,28]. In this case, the movement of temporally fated cells from the ventral leaflet of the optic vesicle is required to compact the future nasal domain, thereby defining the relative sizes of these two axes. Thus, patterning of the NT axis is intimately linked with eye morphogenesis.

Meis1-depleted embryos display defects in NT patterning, especially with regard to the specification of the temporal retina. The early expression of *foxd1*, an essential regulator of temporal identity, is downregulated in *meis1 *morphants (Figure 6F, G). Furthermore, the DV position of *foxd1*-expressing cells is altered in Meis1-depleted embryos compared to equivalently staged wild-type embryos. By 15 hpf, *foxd1*-expressing cells have largely moved from the ventral optic vesicle leaflet to the dorsal leaflet in wild-type embryos. However, in *meis1 *morphants, *foxd1*-expressing cells are still located in the ventral leaflet, suggesting that temporal cell movements into the neural retina are impaired in Meis1-depleted embryos. Foxd1 is known to regulate *ephA *expression in the temporal retina [20]. Since Ephs and Ephrins are involved in cell sorting and adhesion [86], the decrease in temporal *epha3*, *epha4b*, and *epha7 *expression (Figure 8; Additional file 7) is likely to alter cohesive cell behaviours amongst temporal retinal progenitors and may contribute to hampered cell movements in *meis1 *morphants.

We also observe a subtle expansion of Fgf signalling in the optic vesicle of *meis1 *morphants (Figure 7A-H') that could contribute to the NT patterning defects. Indeed, a partial inhibition of Fgf signalling in *meis1 *morphants is sufficient to restore temporal identity to at least wild-type levels (Figure 8). However, the nasal-to-temporal shift in axial identity caused by FgfR-inhibitor treatment is not as robust in Meis1-depleted embryos as in uninjected controls. As such, these data suggest that expanded Fgf signalling is unlikely to be the only contributing factor to the NT patterning defects in Meis1-depleted embryos. The balance between nasal and temporal identity is mechanistically complex and interdependent [28], and the early expansion of Fgf signalling and *foxg1a *expression in 15-hpf *meis1 *morphants could be a consequence, rather than a cause, of decreased temporal identity. Consistent with this idea is the observation that temporal identity is robustly reduced in *meis1 *morphants at both 15 hpf and 28 hpf (Figure 6F-J; Additional file 7), whereas the expansion of nasal identity and Fgf signalling is more variable at these same stages (Figures 6A-E and 7). Furthermore, we do not observe any consistent change in the expression of *fgf3*, *fgf8*, or *fgf24 *between the stages of 12 and 15 hpf (data not shown). It is currently unknown if the temporal retina is actively specified, and future studies will determine whether Meis1 plays a specific role in regulating temporal retinal identity.

## Conclusions

In this work, we demonstrate that the homeodomain transcription factor Meis1 plays an important role in axial patterning of both the retina and tectum, thereby contributing to retinotectal map formation in zebrafish. We describe a novel role for Meis1 in the positive regulation of retinal Bmp signalling by activating *smad1 *transcription and repressing *fsta *expression. Additionally, Meis1 plays an important role in specifying temporal identity in the retina. Lastly, Meis1 positively regulates the expression of *ephrin *genes in the tectum. In summary, Meis1 functions to specify positional information throughout the visual system to support the retinotopic organization of ganglion cell axons on the optic tectum.

## Materials and methods

### Fish lines and maintenance

Adult fish were cared for according to standard protocols [87]. The AB strain of wild-type fish was used for all experiments. Embryos were grown at either 25.5°C, 28.5°C, or 33°C and staged according to Kimmel *et al. *[88]. Embryos that were analyzed past the stage of 24 hpf were grown in embryo media supplemented with 0.003% 1-phenyl 2-thiourea (PTU; Sigma-Aldrich, St. Louis, MO, USA) to prevent pigment formation. We find that the *meis1 *morpholino causes a small delay in development (approximately 1 hour younger at 24 hpf). We have used somite counts, tail curvature and eye morphology to equalize stages between uninjected controls and morphants, and have analyzed multiple developmental stages to demonstrate that the morphant phenotypes do not reflect a case of developmental delay.

### Morpholinos, mRNA injections, and Fgf receptor inhibition

All morpholinos were purchased from Gene Tools (Philomath, OR, USA) and dissolved to their working concentrations in Danieau buffer. The *meis1 *[44], *gdf6a *[89], and *smad5 *[83] morpholinos have all been described previously. The second, non-overlapping *meis1 *morpholino (*meis1*NOL: CCCTCCACACTCCCTCGTCTTCCTT) was used at a concentration of 2 mg/ml and approximately 2 nl was injected into one-cell embryos. *fsta*, *GDF6*, and *myc-meis1 *mRNAs were synthesized from NotI linearized CS2-*fsta *[90], CS2-*GDF6 *[91], and CS3+mt-*meis1 *[38] templates, respectively, using the SP6 mMessage Machine kit (Applied Biosystems/Ambion, Austin, TX, USA) and purified using Microcon YM-50 columns (Millipore, Billerica, MA, USA). Embryos were injected at the one-cell stage with 10 pg of *GDF6 *mRNA, 100 pg of *myc-meis1 *mRNA or 200 pg of *fsta *mRNA. Inhibition of Fgf signalling was done using PD173074 (Stemgent, Cambridge, MA, USA), a small molecule ATP-competitive inhibitor of FgfRs [92]. Wild-type and *meis1 *morphant embryos were incubated in a 50 μM solution of PD173074 or 0.5% dimethyl sulfoxide (DMSO) control between the stages of 90% epiboly and seven somites. The embryos were removed from the treatment dishes, washed three times in embryo media, and grown at 28.5°C until fixation at the 28 hpf stage.

### mRNA *in situ *hybridization

mRNA *in situ *hybridizations were performed essentially as described [93] with the following modifications. Antisense DIG-labelled probes were prepared either from a linearized plasmid template containing a gene-specific insert, or from a gene-specific PCR product containing either a T3 or T7 RNA polymerase site. In either case, each 20 μl probe synthesis reaction included 200 to 400 ng of template, 2 μl of 10× transcription buffer (Roche, Indianapolis, IN, USA), 2 μl 10× DIG RNA labelling mix (Roche), 20 units (0.5 μl) RNAsein (Promega, Madison, WI, USA), 20 units (1 μl) of either T3 or T7 RNA polymerase (Roche), and RNase-free water up to 20 μl. Reactions were performed for 2 hours at 37°C, with another 20 units of the appropriate RNA polymerase was added midway through the incubation. Following a 10-minute DNase treatment at 37°C, reactions were purified using SigmaSpin Post-Reaction Clean-Up Columns (Sigma-Aldrich) following the manufacturer's protocol. RNAlater (10 μl; Sigma-Aldrich) was added to the flowthrough and the resultant probe was diluted 1:300 in hybridization solution and stored at -20°C. All steps of the protocol involving embryos were performed in 1.7 ml microfuge tubes. Pre-hybridization, hybridization, and wash steps were carried out in a 65°C waterbath. The 65°C washes were done as follows: 1 × 5 minutes for each of three solutions - 66% hybridization solution/33% 2× SSC, 33% hybridization solution/66% 2× SSC, and 100% 2× SSC/0.1% Tween-20; high stringency washes were done for 1 × 20 minutes in 0.2× SSC/0.1% Tween-20 and 2× 20 minutes in 0.1× SSC/0.1% Tween-20. At room temperature, 1 × 5 minute washes were done with the following solutions: 66% 0.2× SSC/33% phosphate-buffered saline Tween-20 (PBST); 33% 0.2× SSC/66% PBST; 100% PBST. Embryos were incubated for at least 2 hours in blocking solution and then incubated overnight at 4°C in a 1:5,000 dilution of sheep anti-DIG-AP FAB fragments (Roche) in blocking solution. Embryos were washed 5 × 15 minutes in PBST at room temperature to remove the antibody. The colouration reaction was performed using either the standard NBT/BCIP reagents dissolved in Alkaline Tris colouration buffer, or with BM Purple (Roche). For BM Purple colouration, embryos were rinsed twice briefly with water following the PBST washes to remove salt before the addition of 500 μl of BM Purple colouration solution. To photograph the embryos, the yolk was manually removed and the embryos were equilibrated in 50% and 70% glycerol solutions before being mounted. Embryos were photographed on a Zeiss AxioImager.Z1 scope with an Axiocam HRm camera with RGB filters. Embryos still on the yolk were photographed using a Zeiss Discovery.V8 stereoscope fitted with a QImaging micropublisher camera. All figures were assembled in Photoshop.

### Retinal ganglion cell labelling

To analyze RGC axon mapping, axons were labelled with two lipophilic dyes (DiI, 1,1', di-octadecyl-3,3,3'3'-tetramethylindocarbocyanine perchlorate; DiO, 3,3'-dioladecyloxacarbocyanine perchlorate) and their termination zones visualized using a Leica TCS-SP2 confocal microscope. DiI was dissolved in dimethylformamide at a concentration of 25 mg/ml, while DiO was dissolved in chloroform at a concentration of 25 mg/ml. For visualization of nuclei, embryos were incubated in Hoechst 33258 stain (Invitrogen, Carlsbad, CA, USA) at 2.5 μg/ml in PBS/0.0005% Tween-20) overnight at 28.5°C. Efforts were made to inject a lower volume of dye into *meis1 *morphants to account for their reduced eye size. To ensure that any overlap in the RGC termination zones we observed was not due to dye bleeding in the retina, labelled eyes were imaged to confirm the accuracy of the injections, and any embryos that did not have distinct red and green RGC axon tracts leaving the retina were excluded from further analysis.

### Immunohistochemistry

For immunohistochemistry, the following primary antibodies and dilutions were used: anti-Meis1 P2A6-1 mouse monoclonal 1:5 [94]; anti-Phospho-Smad1/5/8 rabbit polyclonal 1:200 (Cell Signaling, Danvers, MA, USA); 1:500 anti-acetylated tubulin 6-11B-1 mouse monoclonal (Sigma-Aldrich). Secondary antibodies were either anti-mouse or anti-rabbit Alexa Fluor 488 (Invitrogen) diluted to 1:1,000. For the anti-Meis1 P2A6-1 and anti-Phospho-Smad1/5/8 immunos, standard 4% paraformaldehyde fixation and 10 μg/ml protK permeabilization protocols were used. The antibodies were diluted in 1× PBS/0.1% Triton-X/1% bovine serum albumin/10% goat serum blocking solution. For the acetylated tubulin stains, embryos were fixed overnight at 4°C in Dent's fixative (80% methanol/20% DMSO), gradually rehydrated, washed 3 × 5 minutes in 1× PBS/0.5% Tween-20, and blocked in 1× PBS/0.5% Tween-20/1% DMSO/1% bovine serum albumin/10% goat serum. To mark nuclei, Hoechst 33258 (Invitrogen) at a concentration of 10 μg/ml was included in the overnight secondary antibody incubation step. All images were taken on either a Leica TCS-SP2 or a Zeiss LSM 510 confocal microscope. Z-projections were made in ImageJ and figures assembled in Photoshop.

### Gene expression profiling and quantification of tectal neuropil size

The method of profiling of retinal mRNA *in situ *hybridizations was based on that described by Picker and Brand [27] with the following modifications. The mRNA *in situ *hybridization data used for quantification were all from a single round of morpholino injections and/or pharmaceutical treatments in order to control for variability in experiment-to-experiment differences in mRNA *in situ *staining intensity. Inverted grayscale images of dissected, flat-mounted retinas were prepared and oriented in Photoshop. The images were imported to ImageJ for pixel intensity analysis using the Oval profile plugin. Using the 'Along Oval' analysis mode, the pixel intensity was determined for 360 points around the circumference of the eye and these values were exported to Microsoft Excel for analysis and graphing. Two series of measurements were made per eye: one proximal to the lens and the other more distal. These two series were averaged to arrive at a single 360° series of pixel intensities per eye. Using these averaged values, the nasal (n) and temporal (t) positions (in degrees) where pixel intensity fell to the halfway point between its minimum and maximum values (nmax/2° and tmax/2°) were determined for each eye. For the *vax2 *and *ephb2 *probes, the ventro-nasal and ventro-temporal regions (separated by the choroid fissure) were treated as separate domains, each with their own maximum pixel intensity value. For each *in situ *probe, the means of the wild-type nmax/2° and/or tmax/2° were compared to the corresponding means for the *meis1 *morphant eyes using an unpaired, two-tailed *t*-test using a *P*-value of 0.01 as the cutoff for significance. The resulting mean nmax/2° and tmax/2° values were graphed using the Doughnut chart in Excel. The number of eyes used for each analysis along with the mean nmax/2° and/or tmax/2° values (± one standard deviation) are provided in the tables accompanying the graphs.

To quantify tectal neuropil area in wild-type and *meis1 *morphant embryos, images of Hoechst 33258 stained 5-dpf embryos were analyzed in ImageJ. The nuclei-free area of the neuropil were selected freehand and the pixel area was calculated using the Measure function. Measurements were compiled and graphed in Excel and the mean area values for wild-type and *meis1 *morphant embryos were compared using an unpaired, two-tailed *t*-test.

## Abbreviations

Bmp: Bone morphogenetic protein; DMSO: dimethyl sulfoxide; DV: dorsal-ventral; *efn*: *ephrin*; Fgf: Fibroblast growth factor; FgfR: Fgf receptor; Fsta: follistatin a; Gdf: Growth differentiation factor; hpf: hours post-fertilization; NOL: non-overlapping; NT: nasal-temporal; PBST: phosphate-buffered saline Tween-20; RGC: retinal ganglion cell.

## Competing interests

The authors declare that they have no competing interests.

## Authors' contributions

TE performed all experiments except for the ganglion cell axon labeling that was done by CRF. TE and AJW conceived of the study, designed the experiments and analyzed the data. TE drafted the manuscript with editorial assistance from CRF and AJW. All authors read and approved the final manuscript.

## Supplementary Material

Additional file 1 **Two independent *meis1 *morpholinos result in similar phenotypes**. **(A-D) **Two independent *meis1 *translation blocking morpholinos effectively knockdown Meis1 protein, as shown by whole mount immunohistochemistry using a Meis1 monoclonal antibody. Hoechst 33258 stain marks the nuclei. **(E-H) **The *meis1 *non-overlapping (NOL) morpholino gives similar phenotypes as the ATG-morpholino (compare with Figure 5A-D and Figure 6F, G). *meis1*NOL morphants exhibit reduced *foxd1 *expression in the presumptive temporal retina (*n *= 27/29) (E, F), and upregulated *fsta *expression in the eye at 13 hpf (*n *= 19/19) (G, H). Dotted lines outline the optic vesicle. Views are dorsal with anterior to the left. Click here for file

Additional file 2 **Meis1-knockdown does not affect patterning of the midbrain-hindbrain boundary**. **(A-F) **mRNA *in situ *hybridization for midbrain-hindbrain boundary (MHB) markers *fgf8a *(A, B), *eng2a *(C, D) and *pax2a *(E, F) in 32-hpf wild-type and *meis1 *morphant embryos. Arrows indicate the relevant gene expression domain at the MHB. The insets in (E, F) are representative dissected eyes showing an upregulation of *pax2a *staining in the optic stalk of *meis1 *morphants (*n *= 18/18). Embryos are co-stained with the hindbrain r3 and r5 marker *egr2b*. Embryos are shown in lateral view with dorsal up and anterior to the left, and the dissected retinas are oriented with dorsal up and nasal to the left. Click here for file

Additional file 3 ***meis1 *and *smad1 *expression in the early optic vesicle**. **(A, B) **mRNA *in situ *hybridizations for *meis1 *(A) and *smad1 *(B) in 10.5-hpf wild-type embryos. The dotted circles indicate the eye fields. Views are lateral with anterior on the top. **(C, D) **Transverse sections of wild-type 13-hpf optic vesicles stained for Meis1 protein (C) and *smad1 *mRNA (D). Note that (C) is the same as shown in Figure 1D. The dotted lines outline the optic vesicle and neural tube. Sections are oriented with dorsal at the top. Click here for file

Additional file 4 **Morpholino-insensitive *myc-meis1 *RNA can rescue the *smad1 *and *fsta *expression defects in *meis1 *morphants**. **(A-H) **mRNA *in situ *hybridizations for *smad1 *(A-D) and *fsta *(E-H) in wild-type (A, E), *myc-meis1 *RNA (B, F), *meis1 *morphant (C, G) and *myc-meis1 *RNA/*meis1 *morphant embryos at 14 hpf. All embryos are shown in dorsal view with anterior to the left. Click here for file

Additional file 5 ***gdf6a *morphants have normal *smad1 *and *fsta *expression at 13 hpf**. **(A-D) **mRNA *in situ *hybridizations for *smad1 *(A, B) and *fsta *(C, D) in wild-type (A, C) and *gdf6a *morphant (B, D) embryos at 13 hpf. Dotted lines outline the optic vesicle. Views are dorsal with anterior to the left. Click here for file

Additional file 6 ***smad5 *expression is normal in *meis1 *morphants**. **(A-D) **mRNA *in situ *hybridization for *smad5 *on wild-type (A, B) and *meis1 *morphant (C, D) embryos at 15 hpf. (A, C) Lateral views with anterior up; (B, D) dorsal views with anterior to the left. Click here for file

Additional file 7 **The temporal expression domains of *epha7 *and *epha4b *are reduced in *meis1 *morphants**. **(A, B) **mRNA *in situ *hybridization (ISH) for *epha7 *on wild-type (A) and *meis1 *morphant (B) embryos at 16 hpf. Arrows indicate the expression of *epha7 *in the presumptive temporal retina. Embryos are shown in dorsal view with anterior to the left. **(C-F) **mRNA ISH for the temporal markers *epha7 *(C, D) and *epha4b *(E, F) in dissected, flat-mounted eyes from 26- to 28-hpf wild-type and *meis1 *morphant embryos. Arrows indicate the dorsal extent of gene expression. Representative dissected eyes are shown. Legend for retinal axial orientation: D, dorsal; V, ventral; N, nasal; T, temporal. Click here for file

Additional file 8 **The RGC axon stalling phenotype in *meis1 *morphants**. **(A, B) **Dorsal-ventral (A) and nasal-temporal (B) RGC axon stalling phenotypes in *meis1 *morphants. Arrows indicate the stalled RGC axons labelled with fluorescent lipophilic dyes DiI (red) and DiO (green). Hoechst 33258 (blue) marks nuclei. All views are dorsal with anterior to the left. Legend for axial position in the tectum: M, medial; L, lateral; A, anterior; P, posterior. Click here for file
