# Supplementary figures and images for: Meis1 specifies positional information in the retina and tectum to organize the zebrafish visual system
Source: Neural Dev. 2010 Sep 1;5:22. doi: 10.1186/1749-8104-5-22 (PMC2939508; doi:10.1186/1749-8104-5-22)

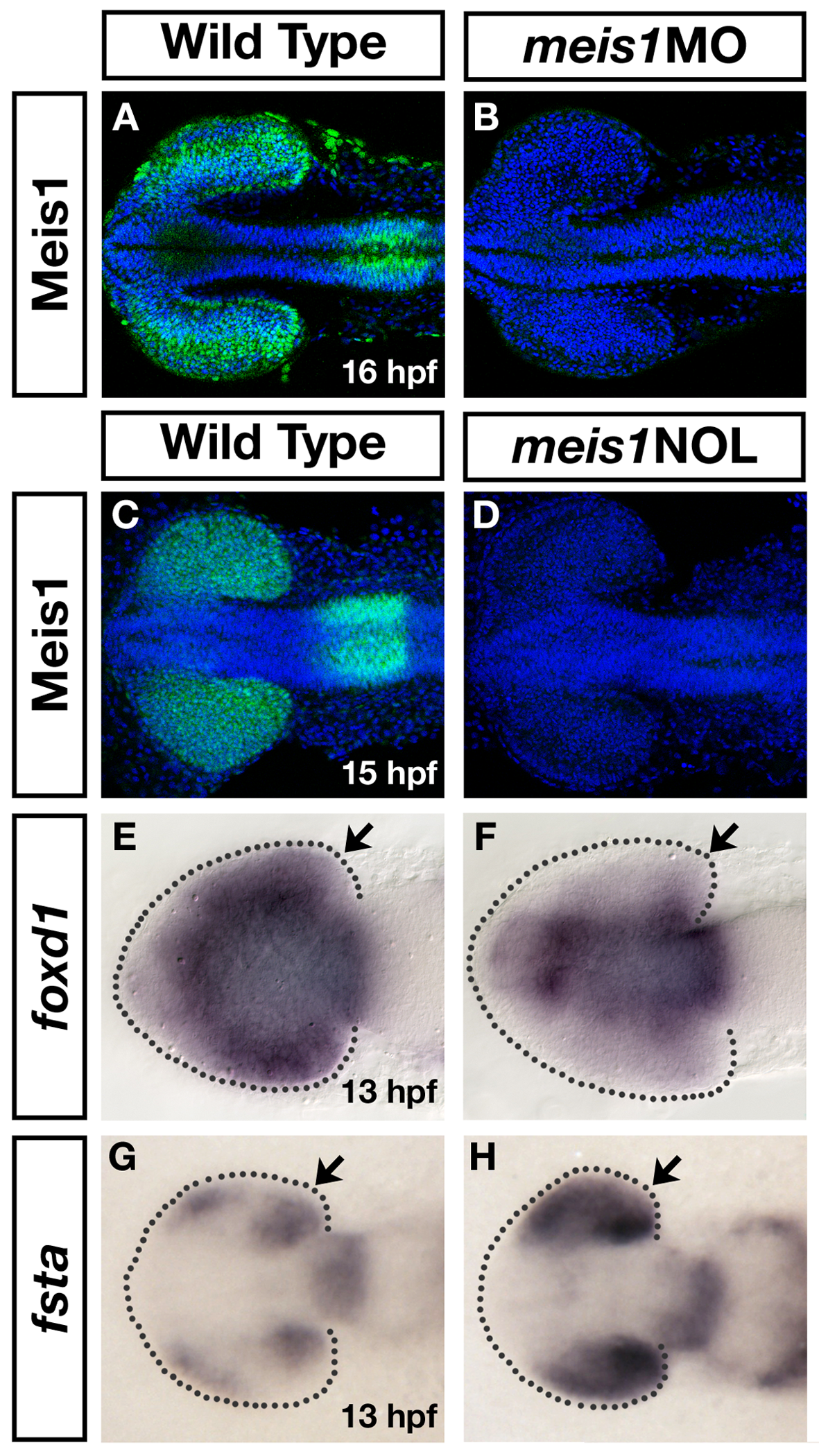

Supplement: Additional file 1 — Two independent meis1 morpholinos result in similar phenotypes. (A-D) Two independent meis1 translation blocking morpholinos effectively knockdown Meis1 protein, as shown by whole mount immunohistochemistry using a Meis1 monoclonal antibody. Hoechst 33258 stain marks the nuclei. (E-H) The meis1 non-overlapping (NOL) morpholino gives similar phenotypes as the ATG-morpholino (compare with Figure 5A-D and Figure 6F, G). meis1NOL morphants exhibit reduced foxd1 expression in the presumptive temporal retina (n = 27/29) (E, F), and upregulated fsta expression in the eye at 13 hpf (n = 19/19) (G, H). Dotted lines outline the optic vesicle. Views are dorsal with anterior to the left. [file 1749-8104-5-22-S1.TIFF]

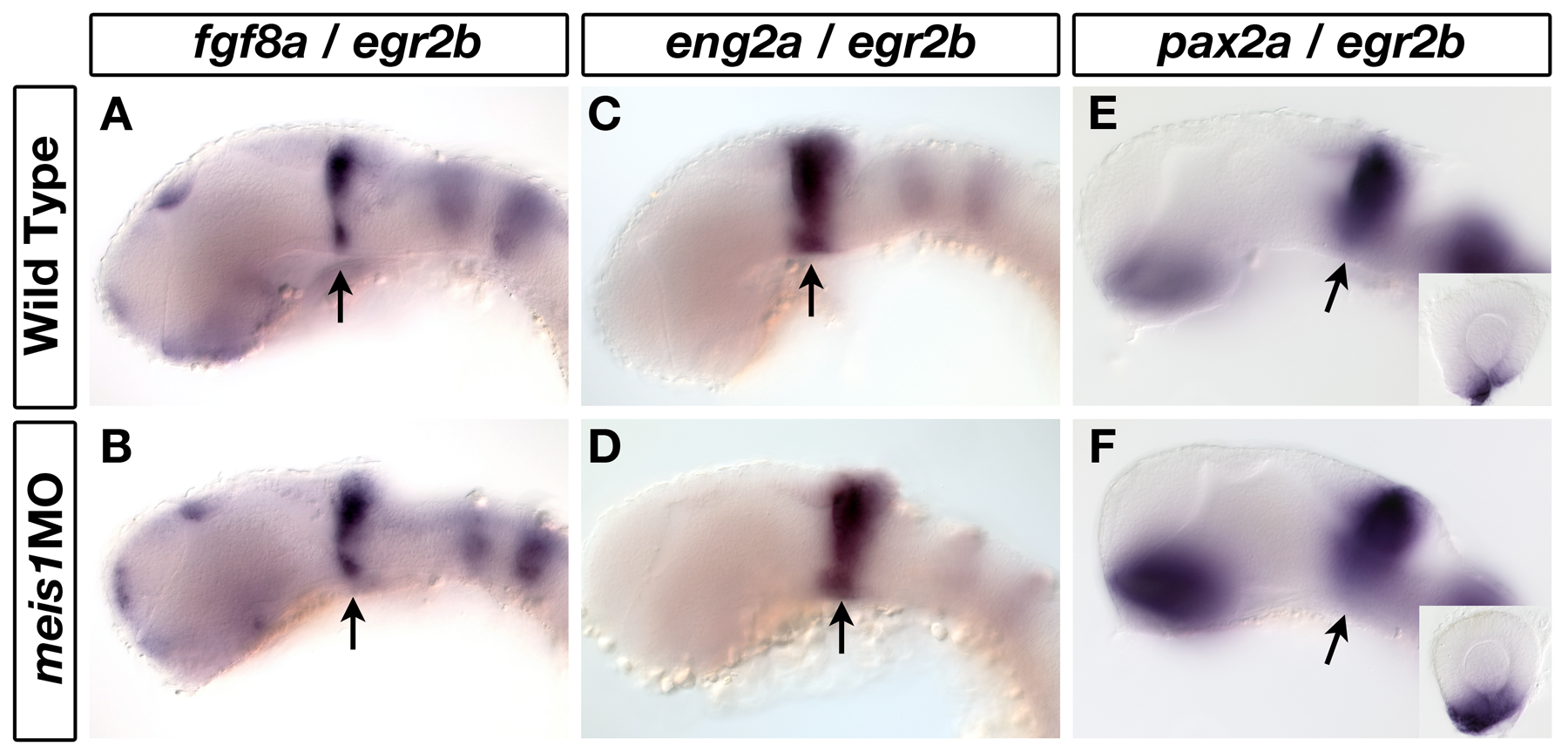

Supplement: Additional file 2 — Meis1-knockdown does not affect patterning of the midbrain-hindbrain boundary. (A-F) mRNA in situ hybridization for midbrain-hindbrain boundary (MHB) markers fgf8a (A, B), eng2a (C, D) and pax2a (E, F) in 32-hpf wild-type and meis1 morphant embryos. Arrows indicate the relevant gene expression domain at the MHB. The insets in (E, F) are representative dissected eyes showing an upregulation of pax2a staining in the optic stalk of meis1 morphants (n = 18/18). Embryos are co-stained with the hindbrain r3 and r5 marker egr2b. Embryos are shown in lateral view with dorsal up and anterior to the left, and the dissected retinas are oriented with dorsal up and nasal to the left. [file 1749-8104-5-22-S2.TIFF]

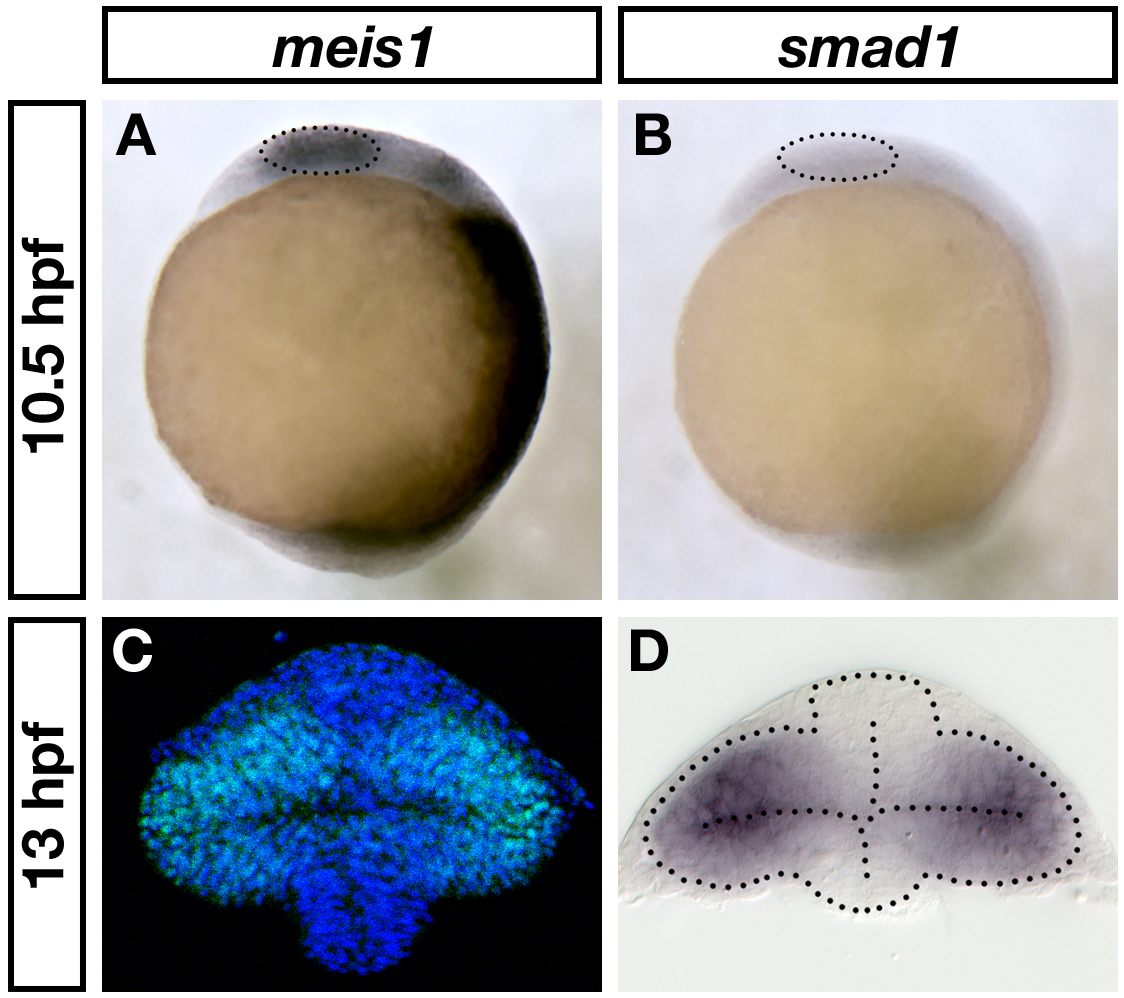

Supplement: Additional file 3 — meis1 and smad1 expression in the early optic vesicle. (A, B) mRNA in situ hybridizations for meis1 (A) and smad1 (B) in 10.5-hpf wild-type embryos. The dotted circles indicate the eye fields. Views are lateral with anterior on the top. (C, D) Transverse sections of wild-type 13-hpf optic vesicles stained for Meis1 protein (C) and smad1 mRNA (D). Note that (C) is the same as shown in Figure 1D. The dotted lines outline the optic vesicle and neural tube. Sections are oriented with dorsal at the top. [file 1749-8104-5-22-S3.TIFF]

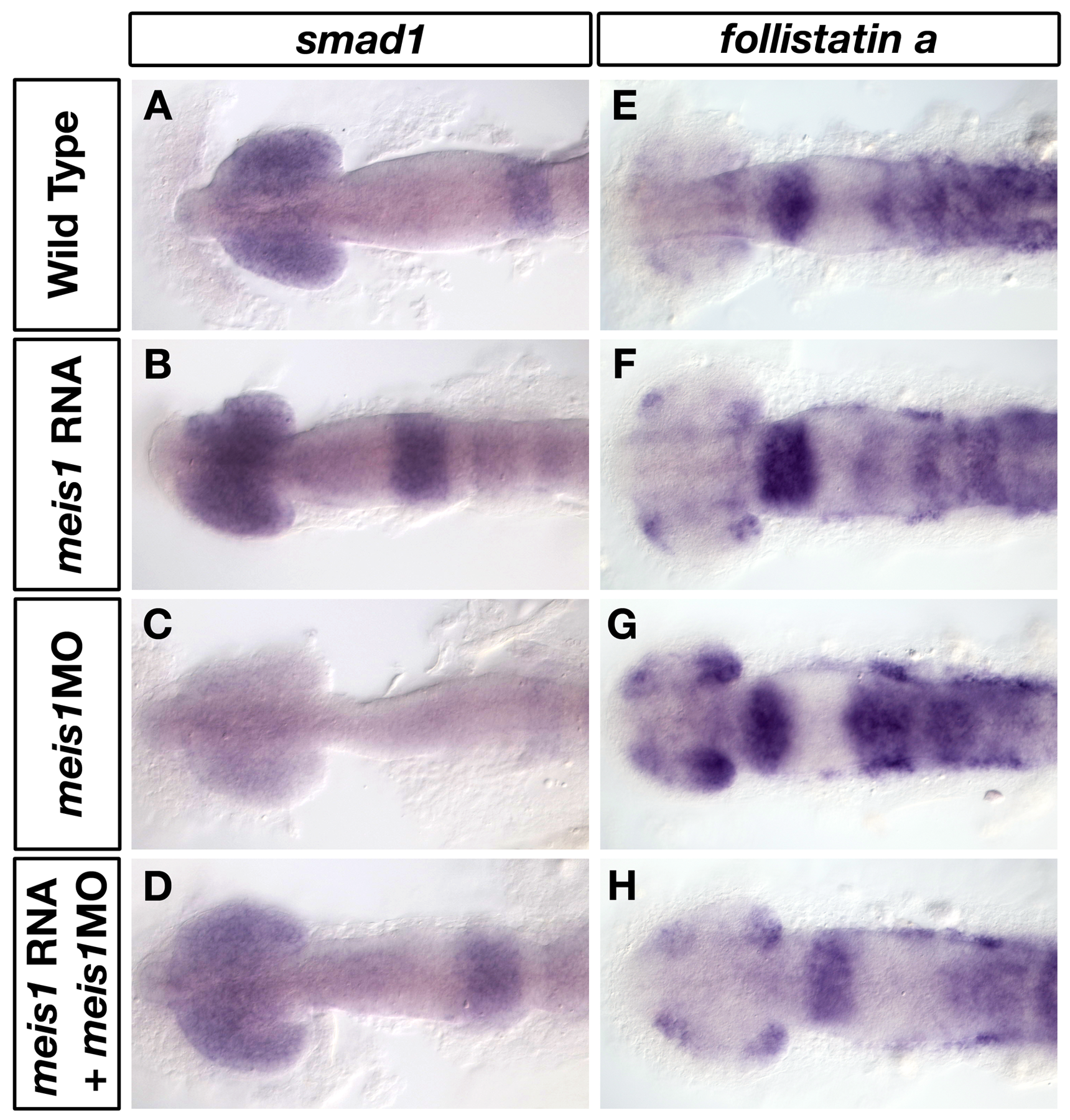

Supplement: Additional file 4 — Morpholino-insensitive myc-meis1 RNA can rescue the smad1 and fsta expression defects in meis1 morphants. (A-H) mRNA in situ hybridizations for smad1 (A-D) and fsta (E-H) in wild-type (A, E), myc-meis1 RNA (B, F), meis1 morphant (C, G) and myc-meis1 RNA/meis1 morphant embryos at 14 hpf. All embryos are shown in dorsal view with anterior to the left. [file 1749-8104-5-22-S4.TIFF]

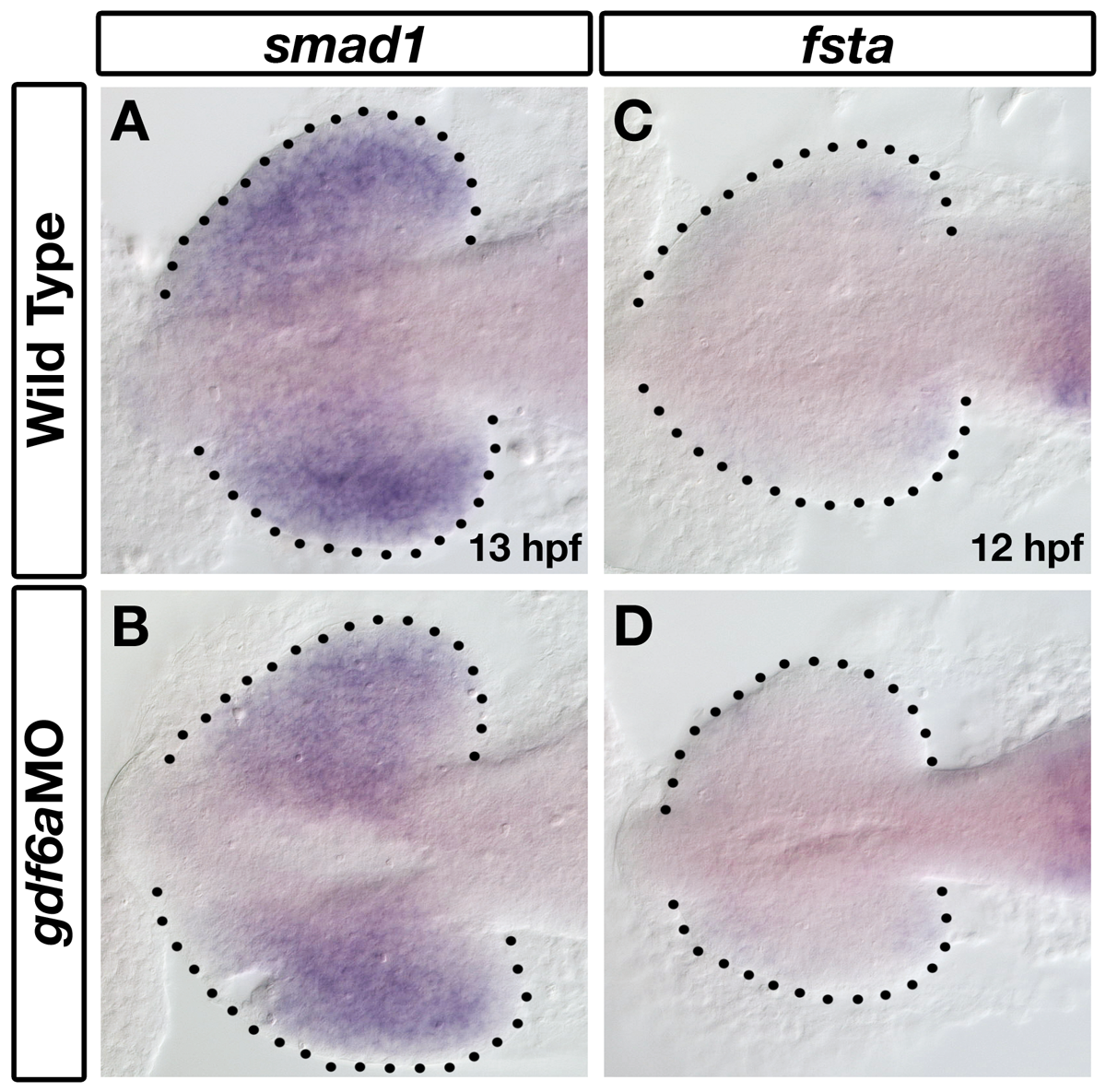

Supplement: Additional file 5 — gdf6a morphants have normal smad1 and fsta expression at 13 hpf. (A-D) mRNA in situ hybridizations for smad1 (A, B) and fsta (C, D) in wild-type (A, C) and gdf6a morphant (B, D) embryos at 13 hpf. Dotted lines outline the optic vesicle. Views are dorsal with anterior to the left. [file 1749-8104-5-22-S5.TIFF]

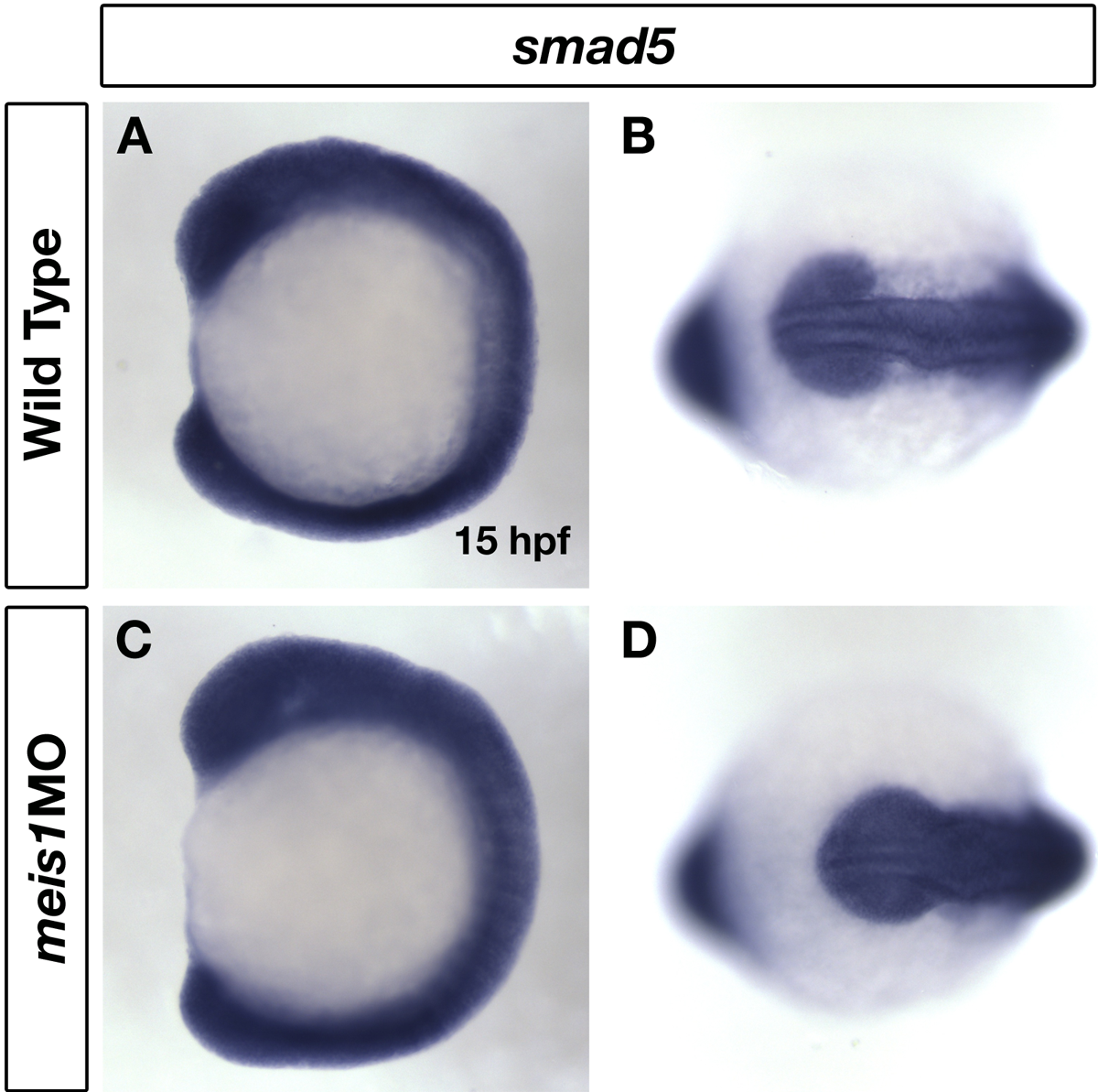

Supplement: Additional file 6 — smad5 expression is normal in meis1 morphants. (A-D) mRNA in situ hybridization for smad5 on wild-type (A, B) and meis1 morphant (C, D) embryos at 15 hpf. (A, C) Lateral views with anterior up; (B, D) dorsal views with anterior to the left. [file 1749-8104-5-22-S6.TIFF]

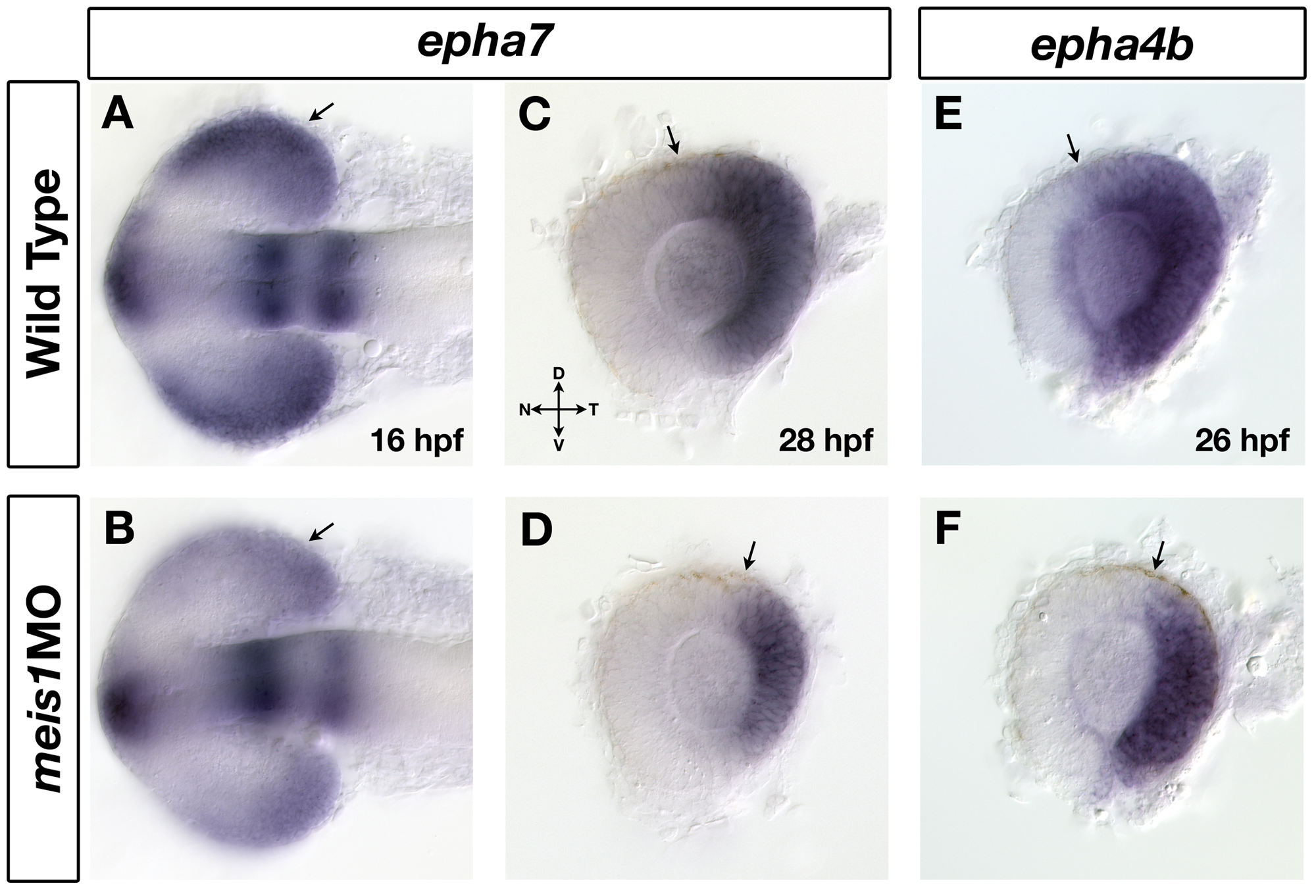

Supplement: Additional file 7 — The temporal expression domains of epha7 and epha4b are reduced in meis1 morphants. (A, B) mRNA in situ hybridization (ISH) for epha7 on wild-type (A) and meis1 morphant (B) embryos at 16 hpf. Arrows indicate the expression of epha7 in the presumptive temporal retina. Embryos are shown in dorsal view with anterior to the left. (C-F) mRNA ISH for the temporal markers epha7 (C, D) and epha4b (E, F) in dissected, flat-mounted eyes from 26- to 28-hpf wild-type and meis1 morphant embryos. Arrows indicate the dorsal extent of gene expression. Representative dissected eyes are shown. Legend for retinal axial orientation: D, dorsal; V, ventral; N, nasal; T, temporal. [file 1749-8104-5-22-S7.TIFF]

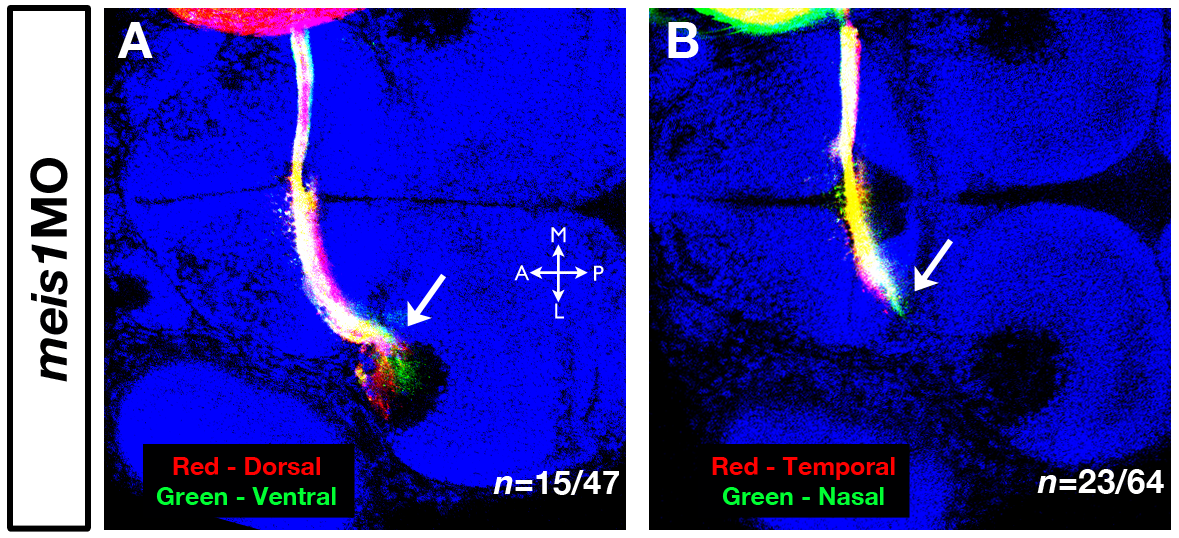

Supplement: Additional file 8 — The RGC axon stalling phenotype in meis1 morphants. (A, B) Dorsal-ventral (A) and nasal-temporal (B) RGC axon stalling phenotypes in meis1 morphants. Arrows indicate the stalled RGC axons labelled with fluorescent lipophilic dyes DiI (red) and DiO (green). Hoechst 33258 (blue) marks nuclei. All views are dorsal with anterior to the left. Legend for axial position in the tectum: M, medial; L, lateral; A, anterior; P, posterior. [file 1749-8104-5-22-S8.TIFF]
